# 4E analysis of a two-stage refrigeration system through surrogate models based on response surface methods and hybrid grey wolf optimizer

**DOI:** 10.1371/journal.pone.0272160

**Published:** 2023-02-03

**Authors:** Rasel Ahmed, Shuhaimi Mahadzir, Adrián Mota-Babiloni, Md Al-Amin, Abdullah Yousuf Usmani, Zaid Ashraf Rana, Hayati Yassin, Saboor Shaik, Fayaz Hussain

**Affiliations:** 1 Chemical Engineering Department, Universiti Teknologi PETRONAS, Seri Iskandar, Perak, Malaysia; 2 Centre for Process Systems Engineering, Institute of Autonomous System, Universiti Teknologi PETRONAS, Seri Iskandar, Perak, Malaysia; 3 ISTENER Research Group, Department of Mechanical Engineering and Construction, Universitat Jaume I (UJI), Castelló de la Plana, Spain; 4 Department of Mechanical Engineering, Universiti Teknologi PETRONAS, Seri Iskandar, Perak, Malaysia; 5 Departement of Mechanical Engineering, Zakir Husain College of Engineering and Technology Aligarh Muslim University, Aligarh, Uttar Pradesh, India; 6 Faculty of Integrated Technologies, Universiti Brunei Darussalam, Jalan Tungku Link, Gadong, Brunei Darussalam; 7 School of Mechanical Engineering, Vellore Institute of Technology, Vellore, Tamil Nadu, India; 8 Modeling Evolutionary Algorithms Simulation and Artificial Intelligence, Faculty of Electrical and Electronics Engineering, Ton Duc Thang University, Ho Chi Minh City, Vietnam; National Institute of Technology Silchar, India, INDIA

## Abstract

Refrigeration systems are complex, non-linear, multi-modal, and multi-dimensional. However, traditional methods are based on a trial and error process to optimize these systems, and a global optimum operating point cannot be guaranteed. Therefore, this work aims to study a two-stage vapor compression refrigeration system (VCRS) through a novel and robust hybrid multi-objective grey wolf optimizer (HMOGWO) algorithm. The system is modeled using response surface methods (RSM) to investigate the impacts of design variables on the set responses. Firstly, the interaction between the system components and their cycle behavior is analyzed by building four surrogate models using RSM. The model fit statistics indicate that they are statistically significant and agree with the design data. Three conflicting scenarios in bi-objective optimization are built focusing on the overall system following the Technique for Order of Preference by Similarity to Ideal Solution (TOPSIS) and Linear Programming Technique for Multidimensional Analysis of Preference (LINMAP) decision-making methods. The optimal solutions indicate that for the first to third scenarios, the exergetic efficiency (EE) and capital expenditure (CAPEX) are optimized by 33.4% and 7.5%, and the EE and operational expenditure (OPEX) are improved by 27.4% and 19.0%. The EE and global warming potential (GWP) are also optimized by 27.2% and 19.1%, where the proposed HMOGWO outperforms the MOGWO and NSGA-II. Finally, the K-means clustering technique is applied for Pareto characterization. Based on the research outcomes, the combined RSM and HMOGWO techniques have proved an excellent solution to simulate and optimize two-stage VCRS.

## 1. Introduction

Global energy consumption is a burning issue nowadays, predicted to increase by 50% in the next forty years [1, 2]. Refrigeration and air-conditioning systems are responsible for around 40% of the total energy consumption [3]. The required energy for residential air conditioners during the summer will increase around 13 times in 2050 and 30 times in 2100, as reported by the Intergovernmental Panel on Climate Change (2014) [4]. In addition, between 50 to 65% of the total electricity consumed in cities is caused by refrigerators and air conditioners [5]. Thus, reducing energy consumption can decrease the dependence on primary energy sources based on fossil fuels and CO_2_ emissions, among other greenhouse gases. Thus, reducing energy consumption in refrigeration can decrease the dependence on primary energy sources based on fossil fuels and CO_2_ emissions, and other greenhouse gases [6]. Among other measures, it is proposed to improve refrigeration systems efficiency by selecting the proper operating conditions [7].

Multi-stage refrigeration is a complex thermodynamic process most widely used in commercial and industrial applications recommended when the pressure ratio is significant. Compared to single-stage cycles, it ensures less power consumption and higher system stability [8]. Given its complex nature, optimization of the process variables is required to enhance the overall system performance. Optimizing vapor compression refrigeration systems (VCRS) has been the focus of many researchers due to their importance.

Nikolaidis and Probert [9] observed in a two-stage VCRS a reduction of irreversibility due to a decrease in exergy loss at the condenser and evaporator. Baakeem et al. [8] optimized a two-stage VCRS using the conjugate direction method. The authors compared the performance of eight different refrigerants based on exergy efficiency, exergy destruction, coefficient of performance (COP), capital expenditure (CAPEX), and operational expenditure (OPEX). Finally, ammonia provided the highest COP of 6.17 and showed the best performance for other measured parameters. Roy et al. [10] investigated the applicability of R32 instead of R410A in a two stage VCRS with a flash intercooler. They optimized the system concerning exergy and total cost using Multi-objectives Genetic Algorithm (MOGA) and observed that R32 performed slightly better for all the criteria. Subsequently, Pak and Ri [11] optimized two-stage vapor compression steam heat pump parameters using a Genetic Algorithm (GA), considering R22, R134a, and R717. They improved the global search ability of GA by implementing a selection method that consists of a protective selection of eugenic and elite individuals and by adjusting the crossover probability according to the population variance and generation numbers. Finally, they successfully minimized the annual cost by 30% and 40% for the two cases. The authors also claimed that their new self-adapting, highly parallel encoding method increased the searchability of GA by 5.5%.

Zhao et al. [12] developed a model-based optimization technique for a single-stage VCRS and applied modified GA for optimization. They implemented this model on a pilot plant and generated experimental data sets for different operating conditions. The authors use the Gray encoding to overcome the limitations of binary representation and store the best populations in each generation. The daily overall energy saving is 8.5%. In another study, Ghorbani et al. [13] proposed a systematic method considering mathematical models and thermodynamic viewpoints to optimize mixed refrigerant cycle parameters. The application of the intelligent algorithm PSO led to better results, achieving 123.09 kW and 11.24 kW more energy savings than non-linear programming (NLP). Zendehboudi et al. [14] performed modeling and multi-objective optimization (MOO) of the R450A single-stage VCRS using RSM and Non-dominated Sorting Genetic Algorithm II (NSGA-II). The authors established the robustness of their designed NSGA II by testing it for various types of objective functions with different complexity. They reduced the motor-compressor electrical power consumption by 18.39% and discharge temperature by 53.31%. They increased the refrigerant mass flow rate by 215.57%.

Wang et al. [15] built an innovative hybrid air conditioning model, combining an ejector with a standard VCRS. The authors applied a hybrid Genetic Algorithm-Enabled Particle Swarm Optimization (PSOGA) algorithm that utilizes the best features of both algorithms, such as fast convergence and high accuracy, respectively. A comparison between PSOGA and on-off control showed that PSOGA could decrease the system’s total energy consumption by 7.36%. Salim and Kim [16] designed a combined power generation consisting of an organic Rankine cycle (ORC), a VCRS, and then applied MOO. For the ORC, they used three dry refrigerants (R245fa, R245ca, and R236ea), and for the VCRS, they used R410A. The authors applied the elitist NSGA II and different weight factors for each decision variable based on their significance. Finally, the maximum efficiency was achieved for R245ca, where the relevant cost is the highest. On the other hand, minimum efficiency was obtained at a minimum cost of R236ea. R245fa successfully gained optimum performance but lay between the two extreme cases.

Conventional optimization techniques consider one variable at a time and analyze the impact of the parameters on the system’s output individually [17]. This process is inefficient because the interaction between multiple variables cannot be analyzed simultaneously [18, 19]. By contrast, the RSM is a statistical technique that overcomes these limitations by allowing users to simultaneously analyze the interaction between multiple independent variables and their effects on the dependent variables using a few experimental data sets [14]. The Central Composite Design (CCD) of RSM is a robust design technique that works better for a small number of data sets, where the built models are not sensitive to missing data. The CCD is also superior to other DOE-based techniques such as Box–Behnken Design, Fractional Factorial Design, Block Design, Quasi-Experimental Design, and the Taguchi Method [20]. By contrast, machine learning techniques such as Artificial Neural Networks (ANN), Kriging, Support Vector Machines (SVM), and hybrid methods work better for large-scale data sets [21].

However, optimizing the refrigeration system’s components, such as compressors and heat exchangers, is a trial and error procedure that requires studying many thermodynamic properties, design rules, empirical knowledge, and calculation [22, 23]. Additionally, the nature of the refrigeration system optimization problem is complex, non-linear, multi-modal, and multi-dimensional [22, 24]. Classic optimization techniques are time-consuming, require gradient information, and may not guarantee the global optima cost-effectively. Therefore, there is always the risk that the designed results are not the optimal ones. This uncertainty leads researchers to apply intelligent optimization algorithms such as GA, PSO, and differential evolution (DE) to refrigeration systems [25, 26]. Subsequently, these algorithms can handle a large amount of data and non-linearities and do not require detailed information about the system and the differentiability of the model. They have superiority over deterministic methods and can generate global or near-global solutions. Nevertheless, because of their heuristic nature, these algorithms sometimes fail to confirm global optima.

The area of metaheuristics is continuously evolving with new, advanced, and efficient algorithms [27]. Grey Wolf Optimizer (GWO) is a novel population-based metaheuristic algorithm that falls under swarm intelligence. This algorithm gained significant attention for applications in every engineering field because of its simplicity, good precision for search, and very few controlling parameters. GWO was successfully applied to optimal power flow [28, 29], parameter estimation [30, 31], feature selection [32–34], wind speed forecasting [35], economic dispatch [36, 37], pattern recognition [38], unit commitment [39, 40], optimal design of photovoltaic arrays [41]. Nonetheless, the basic GWO algorithm suffers from premature convergence in non-linear, non-convex, and multi-modal problems. So, hybrid MOGWO (HMOGWO) utilizes the best features of GWO (strong exploitation ability) and DE (strong exploration capability) algorithms.

Though, multi-objective optimization can represent the optimal realistic scenario of any process by satisfying multiple criteria simultaneously while addressing the trade-off between different objectives. However, to the best of the authors’ knowledge, two-stage VCRS have not been studied for various conflicting objectives of multi-objective optimization, such as EE versus CAPEX, OPEX, and GWP. Additionally, the MOGWO algorithm has not been evaluated by studying and comparing its performance with other metaheuristic approaches for refrigeration system optimization. Therefore, the main goal of this research is to optimize a two-stage VCRS using a novel HMOGWO and demonstrate the robustness and efficiency of the hybrid algorithm for various objectives. The second aim is to analyze the impact of design variables on the considered objectives and model the system using RSM. The purpose is to apply different decision-making methods to determine the optimal design variables and their effectiveness. Finally, the Pareto optimal solution sets are characterized using K-means clustering techniques.

To achieve these goals, the two-stage VCRS is rigorously designed in Aspen HYSYS version 10, and 32 experimental data sets are generated in Design-Expert software based on the bounds of design variables. Each data set is tested in Aspen HYSYS, and four RSM-based surrogate models are constructed from input-output data. Moreover, an HMOGWO with a novel velocity and position update equation was developed in the MATLAB R2019B environment to optimize two different thermo-economic and one thermo-environmental scenario of conflicting bi-objective optimization to ensure the robustness and efficiency of the proposed algorithm. Furthermore, Euclidean non-dimensionalization has been applied, followed by Linear Programming Technique for Multidimensional Analysis of Preference (LINMAP) and Technique for Order of Preference by Similarity to Ideal Solution (TOPSIS) decision-making techniques to select the optimal point on the Pareto front. Their corresponding deviation index is also studied. Finally, the best results of HMOGWO are further compared with the MOGWO and NSGA-II algorithms; here, NSGA-II is the most widely used multi-objective optimization algorithm in refrigeration system research.

## 2. Methodology

### 2.1 System modeling

The base case of the studied two-stage VCRS with a flash chamber is developed based on the concept of Baakeem’s published literature [8] that also followed the general model of two-stage VCRS proposed by Torrella et al. [42]. This general model can be easily adapted to any configuration of two-stage VCRS and is appropriate for a detailed thermo-economic-environmental analysis of the system. In this study, the modeling of a two-stage refrigeration system is based on the following assumptions.

- The system is running at a steady state.
- There are no kinetic or potential energy losses.
- There is no pressure loss between the evaporator and the condenser.
- The refrigerant is saturated when it exits the evaporator and condenser.
- The expansion valve’s throttling operation is isenthalpic.

Table 1 provides the conditions of the base model design and their corresponding sources.

**Table 1:** Base model design parameters.

Parameter	Value	Unit	Reference
Evaporator temperature	0	°C	[8]
Condenser temperature	45	°C	[8]
Outdoor temperature	35	°C	[8]
Indoor temperature	25	°C	
Compressor efficiency	91%	Unitless	[43]
Cooling load	1	kW	[8]
Evaporator heat transfer coefficient (HTC)	0.5	kW m^–2^ K^–1^	[44]
Condenser HTC	0.5	kW m^–2^ K^–1^	[44]
Maintenance factor	1.06	Unitless	[10]
Annual interest rate	14%	Unitless	[10]
Plant lifetime	20	years	[10]
Annual operational hour	4266	hours	[10]
Electrical power cost	0.09	USD kWh^–1^	[45]
Emission factor	0.968	kg kWh^–1^	[45]
Cost of CO_2_e avoided	0.09	USD kgCO_2_e^–1^	[45]
R134a critical temperature	101	°C	[8]
R134a critical pressure	4059	kPa	[8]
R134a ODP	0	Unitless	[8]
R134a GWP_100_	1300	Unitless	[8]

**Fig 1:**
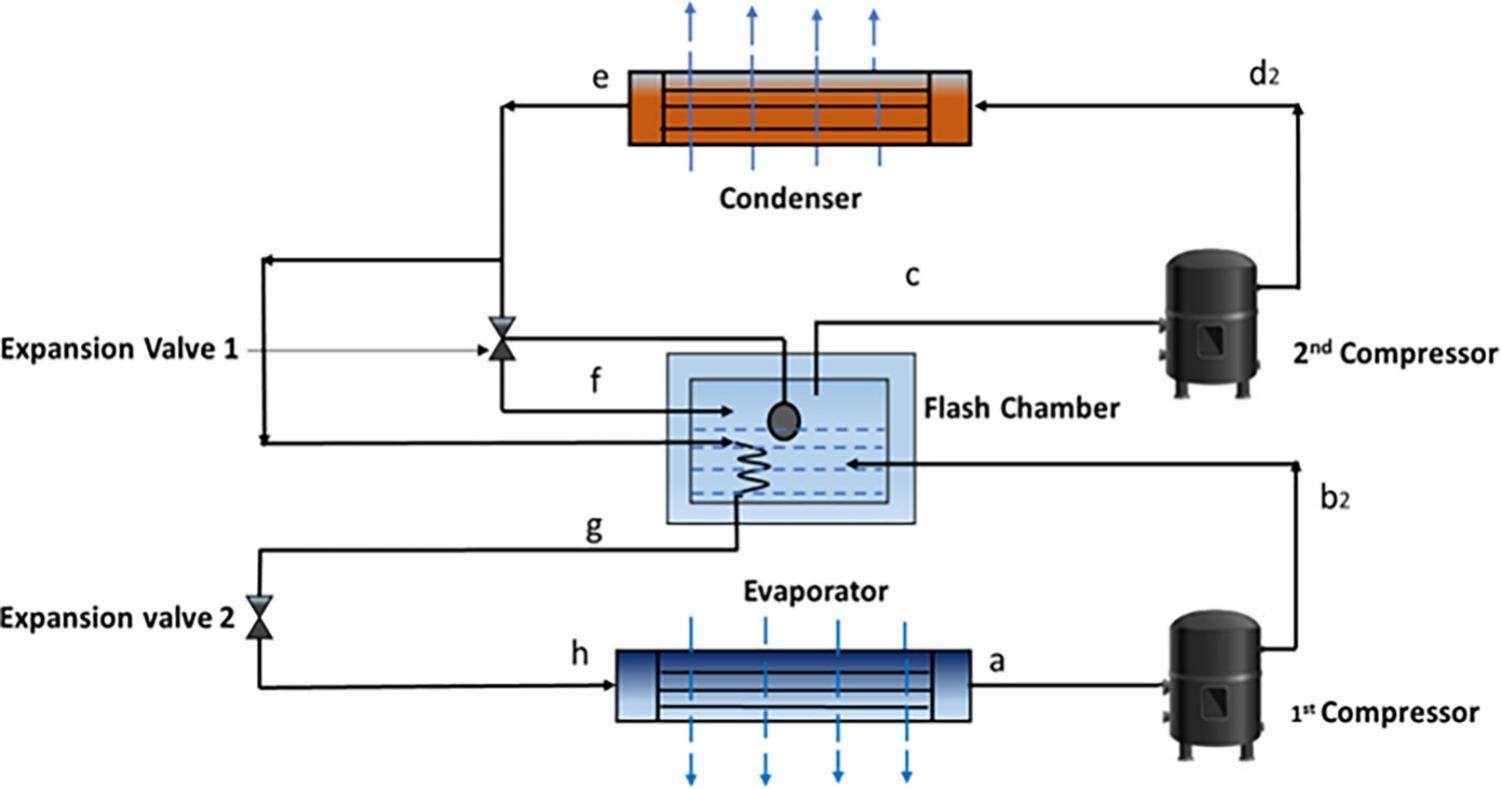
Schematic of the two-stage VCRS with flash inter-cooling.

**Fig 2:**
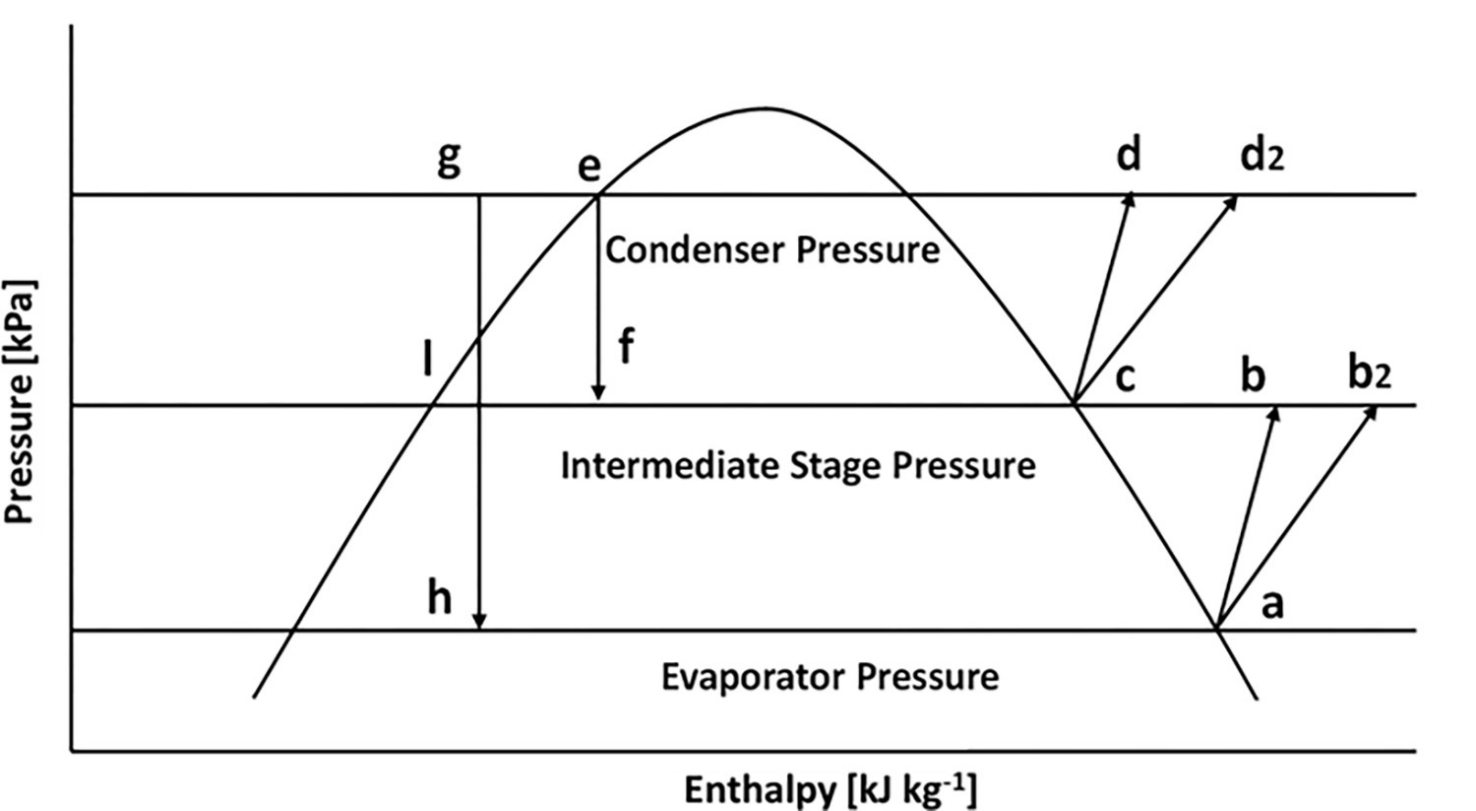
P-h diagram of the designed two-stage VCRS.

The detailed process flow diagram of the two-stage VCRS with flash inter-cooling and its corresponding pressure-enthalpy (P-h) diagram is represented in Figs 1 and 2. Fig 1 shows that the refrigerant R134a leaves the evaporator at the saturated condition at point a. Afterwards, it is compressed by the first stage compressor to an intermediate pressure at point b_2_, where the refrigerant turns into superheated vapor. The superheated refrigerant vapor from b_2_ enters the flash chamber. It is mixed with the cooled refrigerant coming from the condenser. The combined desuperheated refrigerant stream enters the suction of the high-pressure stage compressor at point c and is compressed into superheated refrigerant at point d_2_. The superheated refrigerant loses some enthalpies through point d_2_ to the condenser; the superheated refrigerant loses some enthalpies. After entering the condenser, it cools down to saturated liquid at point e. The refrigerant separates into two streams at the condenser’s outflow point e. A stream passes via an expansion valve before entering the flash chamber. A different stream is first subcooled at point g and then sent via an expansion valve. Because of the throttling action, some refrigerant evaporates when the stream passes through the expansion valve, and vapor is generated at point h. The refrigerant’s mixed vapor and liquid streams enter the evaporator, where the refrigerant provides cooling, and the cycle continues.

### 2.2 Model validation

To validate the studied model, the performance parameters of the designed two stage refrigeration system, including the evaporator temperature, condenser temperature, compressor efficiency, mass flow ratio, plant capacity, COP, total exergy destruction, and exergy efficiency of the designed model, are further compared with the corresponding parameters reported in the literature by Baakeem et al. [44], Table 2. It can be noticed from Table 2 that there is a good agreement between the present model and that developed by Baakeem et al. [44], where the deviations are less than 5% respectively for each index, which further ensures the accuracy of the build model. The modeled refrigeration system reflects the desuperheating and subcooling effect by introducing a flash chamber in the intermediate pressure stage. The intermediate stage pressure is further considered as the basis of this design that relies on the condenser and evaporator pressure and the effectiveness of sub-cooler and compressor parameters [44].

**Table 2:** Validation of the base model.

**Input parameter**		**Values**	
Evaporator temperature [°C]	0		
Condenser temperature [°C]	40		
Indoor temperature [°C]	25		
Outdoor temperature [°C]	35		
Reference temperature [°C]	25		
Evaporator HTC [kW m^–2^ K^–1^]	0.5		
Condenser HTC [kW m^–2^ K^–1^]	0.5		
Compressor efficiency [%]	91		
Cooling capacity [kW]	1		
**Output parameter**	**Baakeem et al.** [44]	**This study**	**Difference**
Mass flow ratio (r)	1.232	1.243	+0.89%
COP	3.61	3.60	-0.27%
Exergy efficiency	33.1%	34.0%	+0.9%
Exergy destruction [W]	117	121	+3.4%

### 2.3 Energy, exergy, economic and environmental (4E) analysis

4E analysis in thermal systems represents the process in terms of thermodynamics, costs, and the environment, which is very important for industrial processes and provides detailed information about the systems and their interactions with economics and environment. The basic thermodynamic equations are used in this study for 4E analysis, which is further explained in the Section 2.4 Response Surface Method (RSM) in S1 File.

RSM is a statistical technique that fits empirical models to experimental data and can be used for process modeling and optimization. It works based on the correlation between the manipulated (independent) variable and the response (dependent) variable [18, 46, 47]. The primary purpose of RSM is to get an optimal response by analyzing the interaction between variables upon evaluating a series of experiments. The main steps of RSM are experimental design, modeling, model testing, finding optimal setpoints, etc., as shown in S1 Fig in the S1 File [19]. RSM can successfully analyze the impact of every single variable and their coupled impact on the process and evaluate the correlation between different design factors and their responses. Therefore, it has been considered a powerful tool widely used for designing, developing, modifying, and optimizing any product or process. In RSM, three types of design are available: and they are a) Central Composite Design (CCD), b) Box Behnken Design (BBD), and c) Optimal (Custom) Design [47]. The CCD is the most widely used and robust technique. Therefore, we applied the CCD for detailed analysis and modeling of the two-stage VCRS.

### 2.4 Hybrid Multi-Objective Grey Wolf Optimizer (HMOGWO)

Swarm intelligence mainly works based on the hunting, searching, flock maintaining mechanisms, and social hierarchy of biological creatures [48, 49]. Grew Wolf Optimizer (GWO) is a novel swarm intelligence algorithm proposed by Mirjalili et al. in 2014 [50]; subsequently, the multi-objective GWO was also proposed by Mirjalili et al. in 2016 [51]. The Grey Wolves (Canis Lupus) are nature’s top predators, having intense prey searching and capturing ability and maintaining social hierarchy and leadership [49]. While most swarm intelligence algorithms advance the search process by following one best solution, the GWO continues by following the three best wolves: alpha, beta, and delta. It makes the GWO less prone to falling into local solutions and premature convergence. The other wolves are called omega wolves, who follow the best three leader wolves. The position has been updated iteratively towards the best position during the search process based on the leader wolves (alpha, beta, delta) positions. The main steps of grey wolf hunting are described below [50].

- Tracking, chasing, proceeding towards the prey.
- Chasing, encompassing, distressing the prey.
- Attacking the prey.

The basic equations of MOGWO are similar to GWO, and the wolves follow the following equation for prey encircling, Eq (1) and (2).


$$\vec{D}=|\vec{C}.{\vec{X}}_{P(j)}-{\vec{X}}_{\left(j\right)}|$$ (1)

$${\vec{X}}_{\left(j+1\right)}={\vec{X}}_{P(j)}-\vec{A}.\vec{D}$$ (2)

$\vec{A}$ and $\vec{C}$ are the randomly generated vectors of coefficients defined below, j is the current iteration number, ${\vec{X}}_{P}$ is the prey position vector and $\vec{X}$ is the position vector of one omega wolves. Here, the position vector refers to some location in the multi-dimensional space of decision variables, which is the search space of the optimum. Coefficient vectors, $\vec{A}$ and $\vec{C}$ are defined as Eq (3) and (4).


$$\vec{A}=2\vec{a}.\vec{r1}-\vec{a}$$ (3)

$$\vec{C}=2\vec{r2}$$ (4)

$\vec{r1}$ and $\vec{r2}$ are random vectors with elements in the range of [0,1] and $\vec{a}$ is a linearly decreasing acceleration constant from [2–0]. The distance of an omega wolf to the best wolves (alpha, beta, and delta) is calculated using the following equations, Eq (5) to (7).


$$\vec{D_{\alpha }}=|\vec{C_{1}}.{\vec{X}}_{\alpha }-\vec{X}|$$ (5)

$$\vec{D_{\beta }}=|\vec{C_{2}}.{\vec{X}}_{\beta }-\vec{X}|$$ (6)

$$\vec{D_{\delta }}=|\vec{C_{3}}.{\vec{X}}_{\delta }-\vec{X}|$$ (7)

$\vec{C_{1}},\vec{C_{2}}\;and\;\vec{C_{3}}$ are random vectors with elements in the range of [0,1], ${\vec{X}}_{\alpha },{\vec{X}}_{\beta }\;and\;{\vec{X}}_{\delta }$ are positions of three leaders (alpha, beta, and delta) wolves, $\vec{X}$ is the current position of all wolves. Eq (5) to (7) determine the distance between the current omega wolves and the leaders; they also indicate the step size of wolves to continue the search. The (omega) wolves typically consider those leader wolves are probably the best position of prey. Accordingly, they update their positions by following leader wolves. Further, they can change their positions through random vectors $\vec{A_{1}},$ $\vec{A_{2}}$ and $\vec{A_{3}}$ as follows, Eq (8) to (11).


$${\vec{X}}_{\left(1\right)}={\vec{X}}_{\alpha }-\vec{A_{1}.}\vec{D_{\alpha }}$$ (8)

$${\vec{X}}_{\left(2\right)}={\vec{X}}_{\beta }-\vec{A_{2}.}\vec{D_{\beta }}$$ (9)

$${\vec{X}}_{\left(3\right)}={\vec{X}}_{\delta }-\vec{A_{3}.}\vec{D_{\delta }}$$ (10)

$${\vec{X}}_{\left(j+1\right)}=\frac{{\vec{X}}_{\left(1\right)}+{\vec{X}}_{\left(2\right)}+{\vec{X}}_{\left(3\right)}}{3}$$ (11)

$\vec{C_{1}},\vec{C_{2}},\vec{C_{3}},\vec{A_{1},}\vec{A_{2},}\vec{A_{3}}$ are random vectors, ${\vec{X}}_{\infty },{\vec{X}}_{\beta },{\vec{X}}_{\delta }$ are positions of three leader wolf alpha, beta, and delta, $\vec{X}$ is the current position of wolves. Eq (5) to (7) determine the distance between the current wolves and the leaders; these equations also indicate the step size of the wolves to continue the search process. The omega wolves update their positions based on Eq (8) to (13). The proposed velocity update equation for the HMOGWO is defined by the following equation.


$$\vec{V_{j+1}}=w.\vec{V_{j}}+\vec{C_{1}}.rand.{\vec{(X}}_{1}-\vec{X})+\vec{C_{2}}.rand.{\vec{(X}}_{2}-\vec{X})+\vec{C_{3}}.rand.{\vec{(X}}_{3}-\vec{X})$$ (12)

Here, $\vec{V_{j}}$ and $\vec{V_{j+1}}$ is the velocity of a wolf at two successive iterations. The following equation defines the modified position update equation concerning the previous position and velocity. In this research, Eq 13 is used for updating the position of each wolf instead of Eq 11.


$${\vec{X}}_{\left(j+1\right)}={\vec{X}}_{\left(j\right)}+\vec{V_{j+1}}$$ (13)

Naturally, the evolutionary operators such as mutation and crossover are known as the most prominent features of nature, which also helps the animal to evolve to a better level. Since the GWO algorithm doesn’t have any evolutionary operator in its original algorithm, this study considers adding the evolutionary operator of DE to the modified GWO to increase the diversity among the wolves and strengthen the search process. Two weight variants are added to the wolves after an individual is chosen to offer variation. The main variation element of DE is the parent difference vector, and each vector includes the parents of a distinct wolf. The mutation operator can be defined as the following equation.


$$W_{j}^{\left(t+1\right)}=X_{3}+F\times (X_{2}-X_{1})$$ (14)

Here 1, 2, and 3 are three separate wolves index numbers from the target vector. W_j_ means the difference vector. The difference vector is (X_2_-X_1_), and F is the scaling factor. A dynamic scaling factor is intended to improve the algorithm’s exploration capacity at the start of the search process to avoid local solutions and strengthen the local search later. The scaling factor is determined dynamically using the following Eq 8.


$$F=f_{min}+(f_{max}-f_{min})\times \frac{Maxit-(it-1)}{Maxit}$$ (15)

Here, it, Maxit, f_min_, f_max_, and F indicates iteration number, maximum iteration number, minimum and maximum values of the scaling factor, and F is the scaling factor. Later, a crossover is done between the target vector *X*_*i*_ and the variation vector $V_{j}^{\left(t+1\right)}$ for producing test wolves $U_{j}^{\left(t+1\right)}$. In the case of crossover, a random crossover technique is applied to confirm that at least one bit of test wolves comes from the variation vector. To determine the other bits, a random number is generated and compared with crossover probability that the following equation can define.

$$\begin{array}{l} U_{j}^{t+1}=W_{j}^{t+1},rand(j)\leq Pc\\ \;=X_{j}rand(j)\geq Pc \end{array}$$ (16)

where j = 1, 2, …… D. X_j_ W_j_, U_j_ are the position, trial and variation vectors, and Pc is crossover probability. The greedy method is applied as the selection procedure. The trial vector is created and compared with the position vector after finishing crossover and mutation, and the best one is selected as the new wolf and replaces the old one.


$$\begin{array}{l} X_{j}^{t+1}=U_{j}^{t+1},f(U_{j}^{t+1})<f(X_{j}^{t})\\ \;=X_{j}^{t},f(U_{j}^{t+1})\geq f(X_{j}^{t}) \end{array}$$ (17)

**Fig 3:**
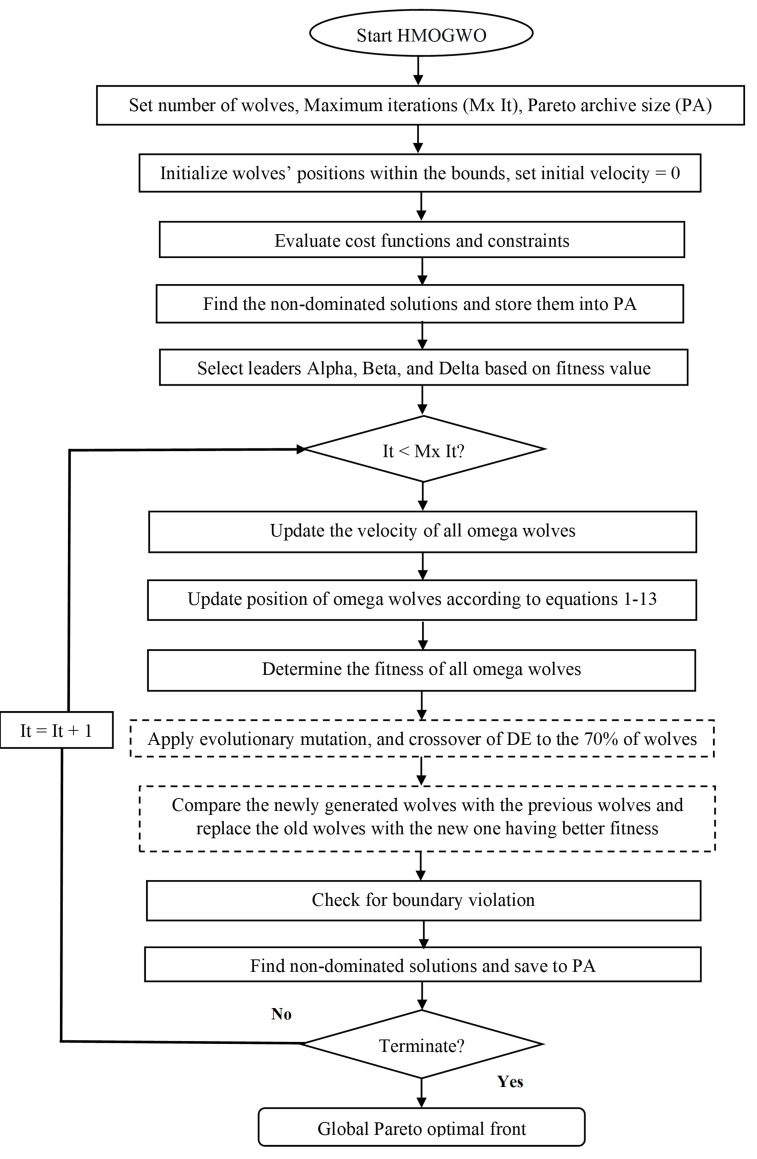
Working principle of HMOGWO.

Here, *t* and *t+1 are two sequential iteration steps*. *X*_*j*_ is the position of a random wolf, and *U*_*j*_ is the trial vector. The space between the wolves is greater at the start of the search, and the mutation operator enhances exploration ability; afterwards, it develops exploitation ability with a smaller distance between the wolves. Fig 3 summarizes the detailed methodology followed in a flow diagram.

In addition to the addressed basic equations of HMOGWO (Eqs 1 to 17), the prominent features that make the proposed HMOGWO efficient to apply to complex MOO problems of two-stage refrigeration systems are described here.

- A novel velocity and position update equation has been developed to improve the searchability of the HMOGWO algorithm.
- The authors incorporated the evolutionary crossover and mutation operators of the differential evolution (DE) algorithm into the basic MOGWO algorithm to strengthen its exploration and exploitation abilities, which aids in avoiding premature convergence and local optima.
- The adaptive parameters a and A allow a smooth transition from global exploration to local exploitation in MOGWO. Whereas the first half of the iterations are dedicated to exploration, the last half is devoted to exploitation.
- The parameter C is generated randomly during the search process to strengthen local optima avoidance.
- The authors applied the roulette wheel selection mechanism to select the leaders from less crowded hypercubes.
- The grid mechanism and the selection leader component are utilized to maintain the diversity in the archive.

### 2.5 Non-dimensionalization and decision making

Non-dimensionalization eliminates the impact of dimensionality on any variables or cost function in the final solution. Otherwise, the solution could be biased or dominated by any specific objectives, which will imbalance the relevant weight or priority of the cost functions. The authors applied the Euclidean non-dimensionalization method to all objective function value matrices in this research. A non-dimensionalized objective can be expressed as Eq (18) [52].


$$F_{ij}^{n}=\frac{F_{ij}}{\sqrt{(\sum_{i=1}^{m}F_{ij}^{2})}}$$ (18)

The $F_{ij}^{n}$ matrix consists of the non-dimensionalized solutions of the Pareto frontier, i and j indicate the solution index and objective index in the objective area. After non-dimensionalization, we apply LINMAP and TOPSIS to find the best and compare their solutions. Using the LINMAP decision-making, an ideal or equilibrium point is fixed where both objectives have their best values. This point is not an expected solution and is not on the Pareto curve. A point that has a minimum distance from the ideal point is considered the best solution. The distance between the ideal solution and any point of the Pareto front is given by Eq (19) [53].


$$E_{di+}=\sum_{j=1}^{m}{\left(F_{ij}-F_{j}^{ideal}\right)}^{2},i=1;\ldots ;n$$ (19)

m represents the objective number, and "i" indicates each solution of the Pareto front, $F_{j}^{ideal}$ is the considered ideal value. In TOPSIS decision-making, ideal and non-ideal solutions are considered, where the non-ideal has the worst fitness value. Two principles are applied when selecting optimum points; the optimum solution has the lowest and highest distances from the ideal point and the highest distance from the non-ideal point. The distance from the non-ideal solution can be represented as Eq (20).


$$E_{di-}=\sum_{j=1}^{m}{\left(F_{ij}-F_{j}^{non-ideal}\right)}^{2}$$ (20)

Consequently, the final form of the TOPSIS decision-making method is represented as Eq (21).


$$Cl_{i}=\frac{E_{di-}}{E_{di+}{+E}_{di-}}$$ (21)

### 2.6 Optimization problem formulation

This section discussed the cost functions, design variables, and relevant constraints of the studied cases. To increase the efficiency of a refrigeration system, the relevant cost will be increased subsequently [54]. This conflicting issue needs to be adequately addressed during the optimization problem formulation, so both can be satisfied simultaneously. This MOO consists of four different and conflicting objective functions, where the first scenario is built to maximize the EE and minimize the CAPEX. Moreover, the second scenario was constructed to maximize EE and minimize OPEX. Likewise, the last scenario is planned to maximize EE and minimize GWP subsequently. The optimization results are represented as a Pareto optimal front so that the designer can choose any point according to each cost function’s weight and importance. The optimization problem has been formulated as Eqs (22) to (25).


$$Min\;F(x)==[f_{1}(x),f_{2}(x)]$$ (22)

Subject to

g(x)≤0 (23)

h(x)≤0 (24)

$$x_{l}<x<x_{u}$$ (25)

Here, f_1_(x) and f_2_(x) represent the cost functions for each case. The design variables are represented as x, and equality and inequality constraints of the problem are indicated by h(x) and g(x). The considered decision variables and their range is presented in Table 3.

**Table 3:** Operating range of the design variables.

Parameter Name	Range	Unit
Evaporator temperature	–30 to 0	°C
Condenser temperature	40 to 60	°C
Intermediate stage pressure	500 to 800	kPa
Refrigerant mass flow rate	0.006 to 0.008	kg s^–1^
Compressor efficiency	0.7 to 0.9	Unitless

The design specification and other properties are described in Table 1.

## 3. Results and discussion

### 3.1 Modelling of the system

The ANOVA test results of the RSM are summarized in Tables 1–4 (in the S2 File Section A). Parameters such as the source, sum of squares, mean of the square, F-value, P-value, significance, and contribution of the factors are included. This test determines the importance of the factors and the interaction between them. Single terms A-E represent the outcomes of changing one factor. The interaction terms (AB-DE) and self-interaction terms A^2^ to E^2^ represent the interaction among the variables and how it affects the dependent variables concurrently. It has been noticed that all of the factors (A-E^2^) have a positive effect on EE, CAPEX, OPEX, and GWP, which indicates a positive correlation between the independent and dependent variables.

**Table 4:** Parameters used by HMOGWO.

Parameters	Value
Grey wolves number	100
Selection	Roulette-wheel
Archive size	100
Maximum Iteration	100
Crossover percentage	70%
F_max_ and F_min_	1.50 and 0.25
a	2 to 0

The authors conducted Fisher’s statistical test to determine the degree of significance for each factor according to their F values [55, 56]. The F-value of the factors that are more than the F-value of the model is significant [55]. The higher F values (108.32 and 254.560, 358.908 and 358.910) represent significant models [55]. According to the F values, the terms A, B, C, E, and A^2^ are significant for EE, whereas the terms A and D are significant for CAPEX analysis. Consequently, the terms A, B, and D are significant for both OPEX and GWP. The probability value (P) indicates the importance of factors, where a value of less than 0.05 indicates that the term is significant with a confidence level of 95% [47]. For the EE analysis, the terms A, B, C, E, BC, A^2^, B^2^, and C^2^ are significant with a contribution of 12.3, 52.7, 11.4, 9.6, 0.5, 10.2, 0.5, and 0.8, respectively. On the other hand, A, B, C, D, E, and AC are noteworthy for the CAPEX, and their corresponding contribution to the overall model is 74.3, 4.6, 5, 10.8, 4.2, and 0.2, respectively. Subsequently, the significant terms for the calculation of OPEX and GWP are similar. They are A, B, C, D, E, AD, AE, A^2^, and C^2^ and their respective contributions are 75.2, 5.7, 4.4, 8.2, 4.3, 0.4, 0.2, 0.1, and 0.8, respectively.

The coefficient of determination (R^2^) is above 0.995 for all four developed models, indicating a perfect fit for the set. These models can explain more than 99.5% of the changes in the variable range. The model’s accuracy is represented by adjusted R^2^, so a higher value of adjusted R^2^ (more than 0.90) is desirable [47]. The adjusted R^2^ value for the EE model is 0.986, 0.994 for CAPEX, and subsequently 0.996 for both the OPEX and GWP models. The higher values of adjusted R^2^ indicate that the models have been developed with significant accuracy. The predicted values of R^2^ are 0.944 for EE, 0.966 for CAPEX, and 0.979 for both OPEX and GWP, which means a good agreement between R^2^, adjusted R^2^, and predicted R^2^. The excellent precision values are standard for all models and indicate adequate signal-to-noise ratios. The EE model shows the lowest STD value of 0.006; the GWP shows the highest STD value among the four models. The coefficient of variation (CV) is the ratio of the standard deviation to the mean value. The CV values are significantly lower and lie between 0.709 and 1.780 for each model.

**Fig 4:**
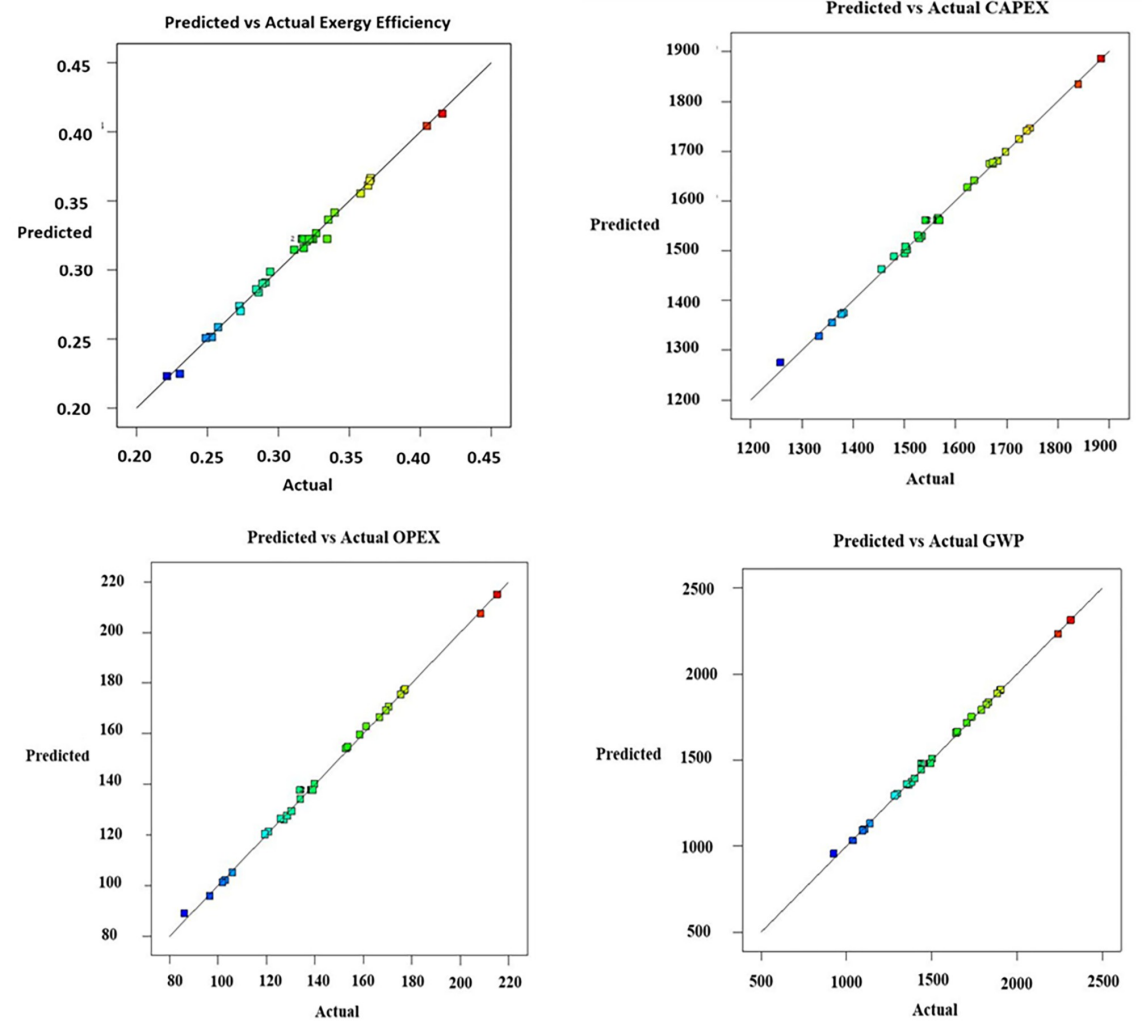
Predicted vs. actual values of a) Exergetic Efficiency, b) CAPEX, c) OPEX, and d) GWP.

From the fit statistics of S4 Table in S1 File, it is noticeable that between the considered seven indexes, the OPEX and GWP models achieved a similar numerical value for four indexes (R^2^, Adjusted R^2^, Predicted R^2^, Adeq Precision). Finally, the P values of the models are small (less than 0.0001), which designates all the models are significant with more than 95% confidence level. The correlation between the designed and predicted parameters for all the models (EE, CAPEX, OPEX, GWP) is represented in Fig 4. It is explicit that the data has been distributed evenly along the line with a minimum percentage of deviations.

### 3.2 Bi-objective optimization results

The optimization scenarios are maximizing EE and minimizing CAPEX of the system, including maintenance costs, maximizing EE and minimizing OPEX, and maximizing EE and minimizing GWP. There is a conflict between the objectives of all three cases, as we need to increase the EE and decrease the other objectives. At the same time, increasing the EE value leads to an increase in the corresponding CAPEX, OPEX, and GWP, making them more challenging to solve and inspiring the authors to design a more robust algorithm. An efficient and robust HMOGWO technique is applied to optimize the conflicting objectives. The corresponding decision variables are evaporator and condenser temperature, intermediate stage pressure, the refrigerant mass flow rate through the evaporator, and compressors’ efficiency. The range of the considered decision variables is mentioned in Table 3. The tuning parameters of the HMOGWO are listed in Table 4.

#### 3.2.1 Results of the first scenario

Employing bi-objective optimization using the HMOGWO technique, the EE and CAPEX of the overall system, including maintenance costs, are optimized concurrently. The quadratic polynomial models of EE and CAPEX are developed by RSM as described in Section 3.1 and have been considered as the cost functions to be optimized. The decision variables presumed for the optimization procedure and the respective upper and lower bounds of the decision variables are described in Section 2.6.

**Fig 5:**
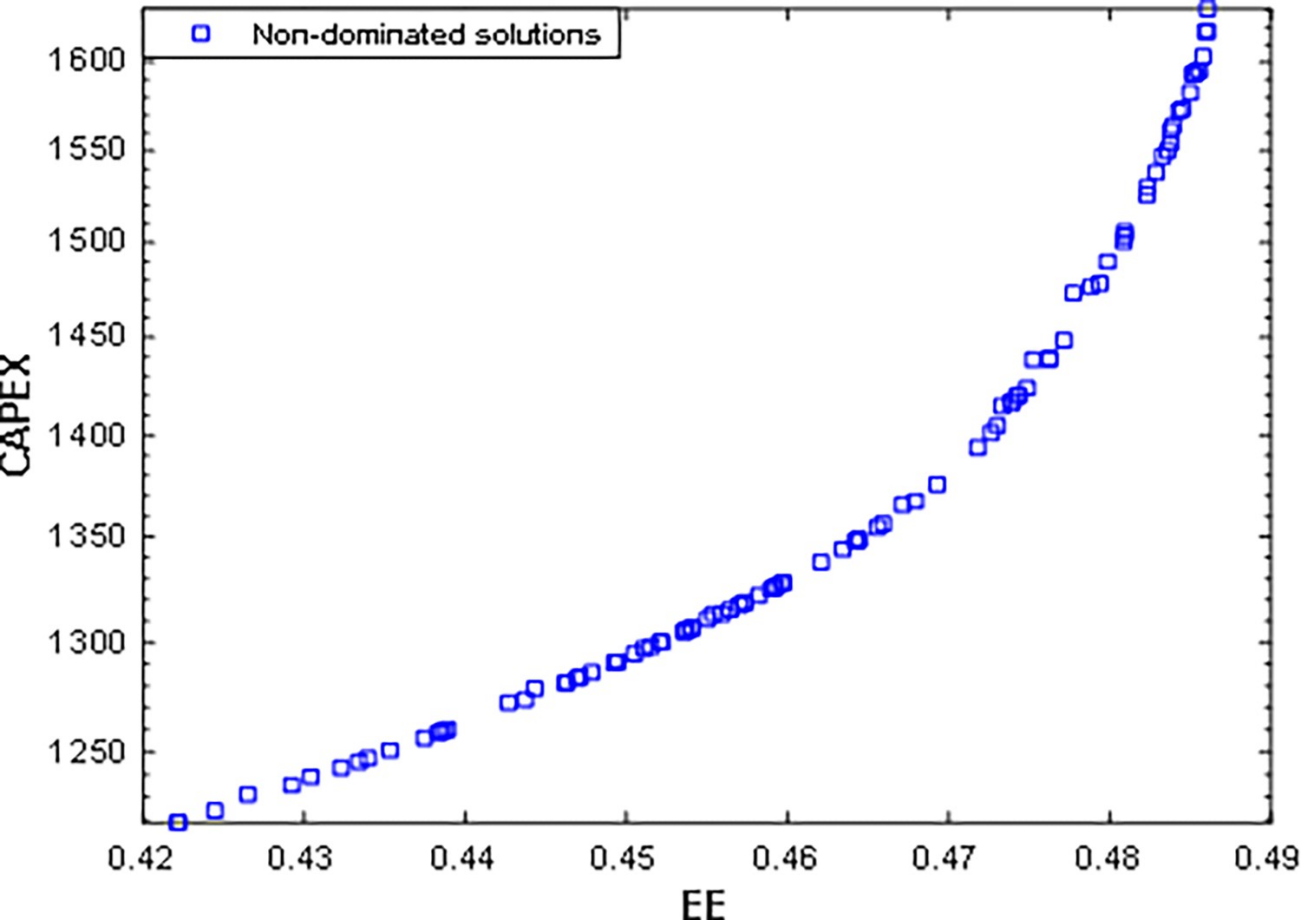
Pareto optimal solutions of the first scenario using HMOGWO.

Fig 5 demonstrates the bi-objective optimization results as a Pareto frontier for the considered two objectives of the first scenario. The conflicting nature of the optimized objectives is visible while increasing the EE also increases the overall CAPEX of the system. In this Pareto frontier, the EE improvement from 42% to 49% caused the corresponding CAPEX increase from 1250 to 1600 (USD per year). The optimum points achieved by the decision-makers are also highlighted in Fig 5.

Additionally, Table 5 summarizes the optimal solutions achieved for the cost functions, decision variables, and the corresponding deviation index for each solution set engaging TOPSIS and LINMAP techniques for the first case, where the LINMAP method shows the minimum deviation for the first scenario. The deviation index indicates the deviation of the achieved optimal solutions concerning both the ideal and non-ideal points, and it can be numerically formulated as indicated in Eqs (26) to (68).


$$Edi_{+}=\sqrt{{\left(EE_{d}-EE_{ideal}\right)}^{2}+{\left(CAPEX_{d}-CAPEX_{ideal}\right)}^{2}}$$ (26)

$$Edi_{-}=\sqrt{{\left(EE_{d}-EE_{non-ideal}\right)}^{2}+{\left(CAPEX_{d}-CAPEX_{non-ideal}\right)}^{2}}$$ (27)

$$Cl_{i}=\frac{{Ed}_{i+}}{{Ed}_{i+}+{Ed}_{i-}}$$ (28)

**Table 5:** Optimal solutions obtained for the first scenario using decision-making methods.

Methods	Decision variables					Objectives		Deviation index
	T_evp_ [°C]	T_con_ [°C]	P_int_ [kPa]	$\dot{m}$ [kg s^–1^]	η_c_	EE [%]	CAPEX [USD per year]	
TOPSIS	–5.28	40	500	0.006	0.9	44.79	1286.29	0.15
LINMAP	–6.74	40	500	0.006	0.9	45.70	1304.97	0.03
Ideal Solution						48.56	1218.519	
Non-Ideal Solution						41.97	1621.081	

**Fig 6:**
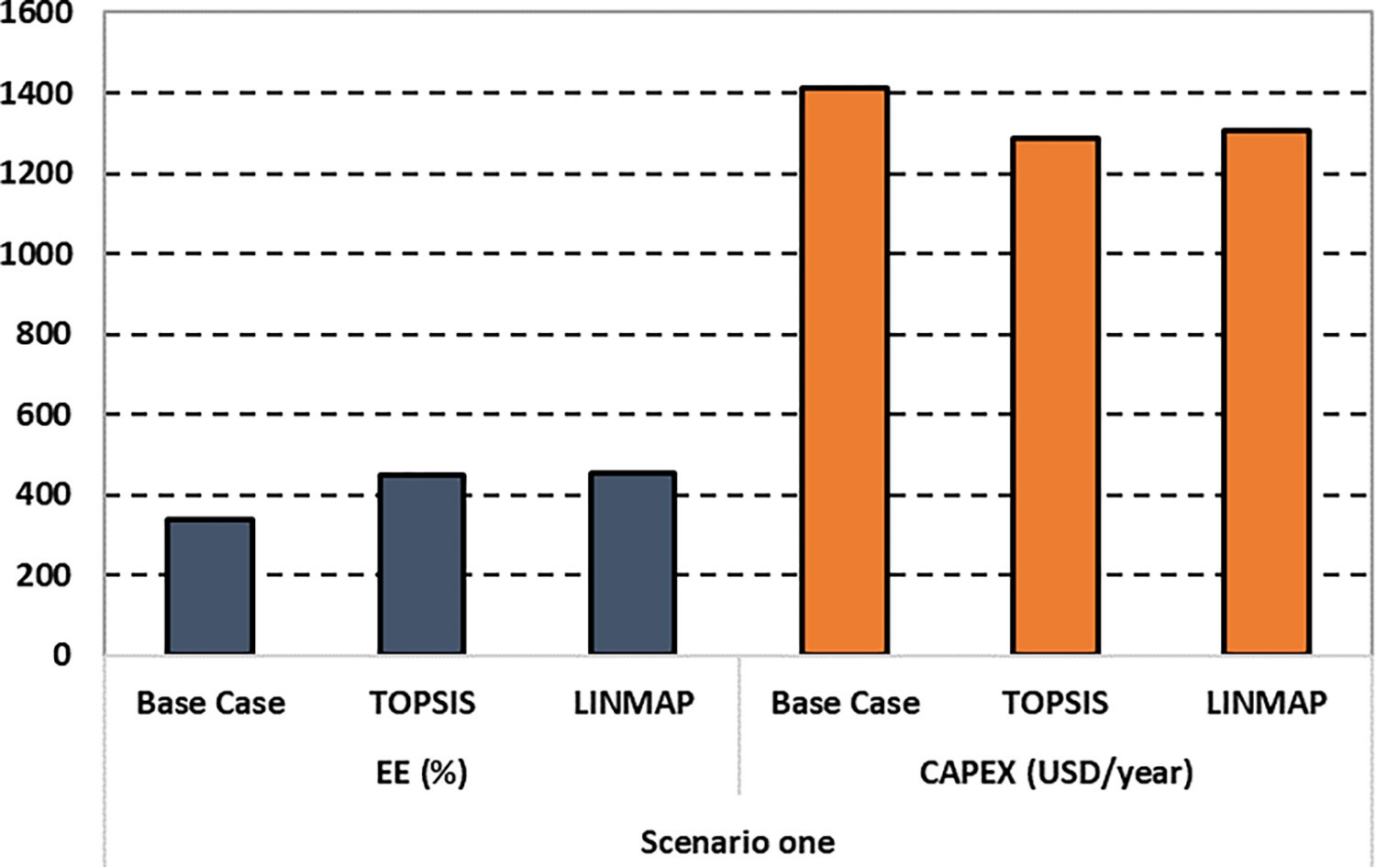
Comparison of EE and CAPEX for the first scenario.

EE_ideal_, EE_non-ideal_, CAPEX_ideal_, and CAPEX_non-ideal_ represent the cost function values at ideal and non-ideal points of the Pareto frontier for EE and CAPEX, respectively. Likewise, EE_d_ and CAPEX_d_ indicate the optimal cost function values achieved by applying the "d" decision-making methods, LINMAP or TOPSIS. Additionally, d_i+_ and d_i-_ represent the Euclidian distance concerning the ideal and non-ideal points, and D is the final deviation index. Fig 6 compares the base case with the result of the first scenario.

#### 3.2.2 Results of the second scenario

**Fig 7:**
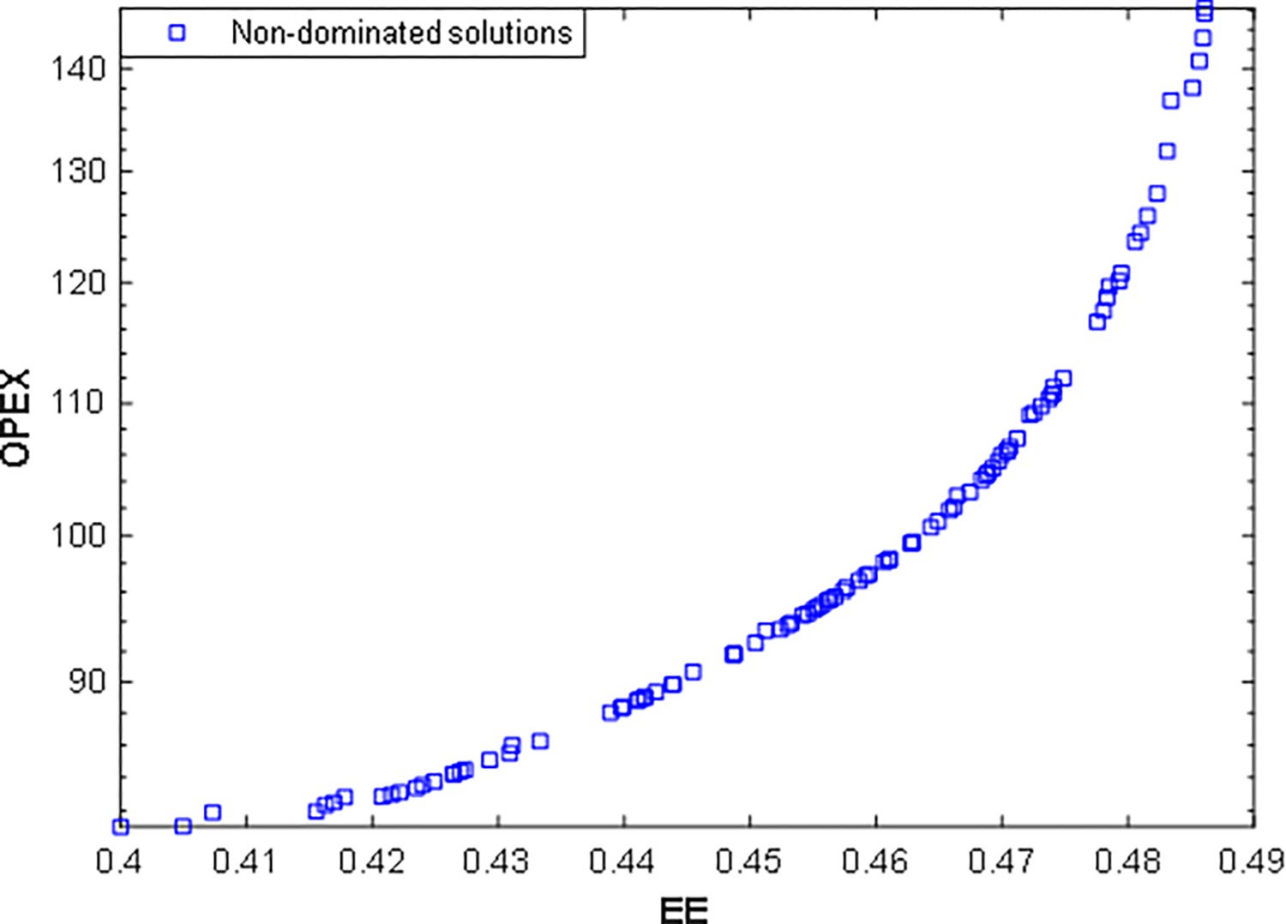
Pareto optimal solutions of the second scenario using HMOGWO.

In this scenario, the EE is maximized, and OPEX is minimized. The quadratic polynomial RSM models of EE and OPEX are considered cost functions to optimize. Fig 7 shows the bi-objective optimization results as a Pareto frontier. The EE varied from 0.40% to 0.49%, and the OPEX from 80 to 148 USD per year.

**Fig 8:**
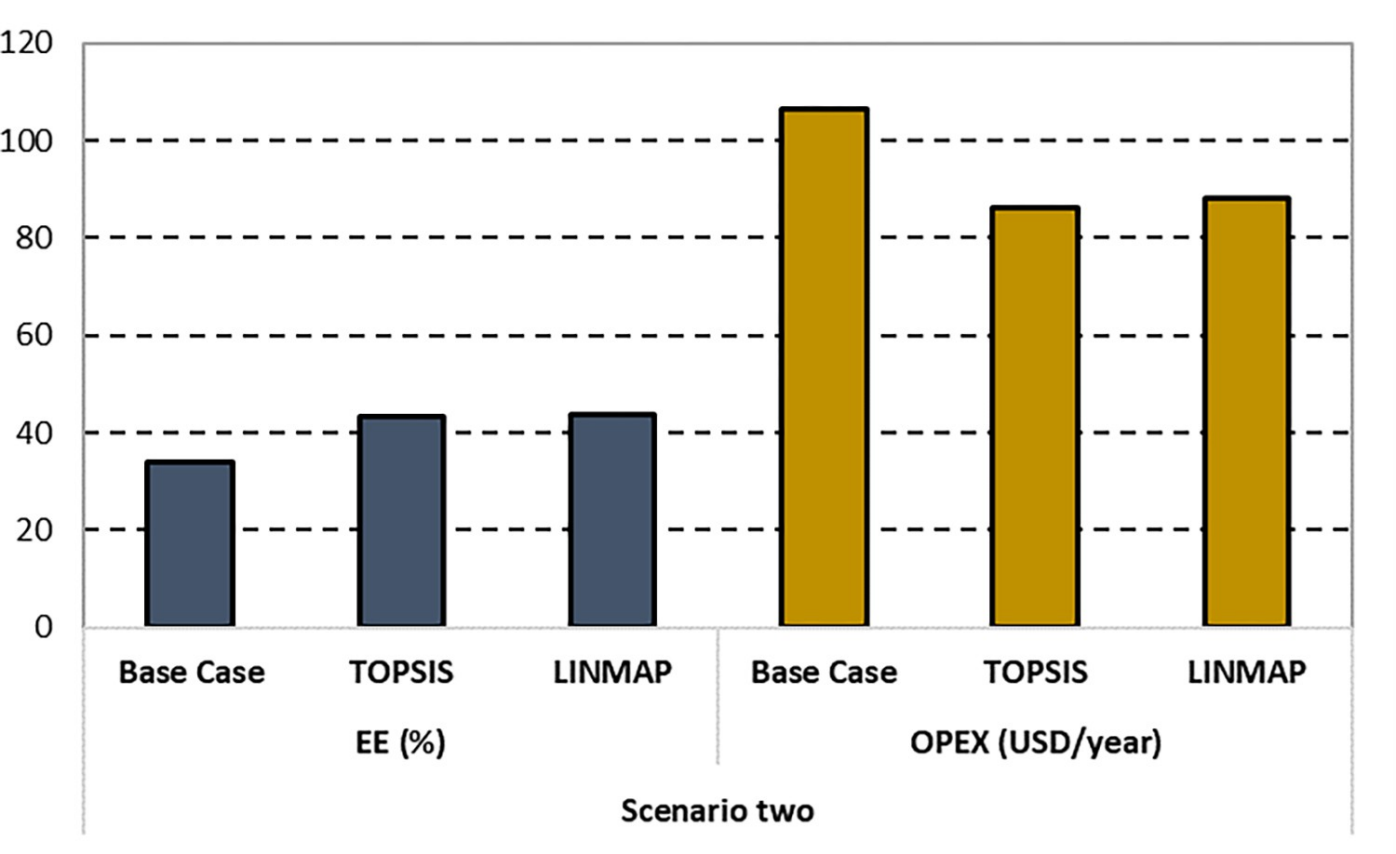
Comparison of EE and OPEX for the second scenario.

Finally, the optimal solutions suggested by the decision-makers, including the cost functions, decision variable values, and the deviation indexes, are summarized in Table 6. The results recommended by TOPSIS show a minimum deviation for the second scenario. Additionally, Fig 8 compares the optimal solutions of the second scenario recommended by both decision-makers with the base case.

**Table 6:** Final solutions obtained via decision-making methods for the second scenario.

Methods	Decision variables					Objectives		Deviation index
	T_evp_ [°C]	T_con_ [°C]	P_int_ [kPa]	$\dot{m}$ [kg s^–1^]	η_c_	EE [%]	OPEX [USD per year]	
TOPSIS	–2.93	40	5	0.006	0.9	43.85	87.06	0.07
LINMAP	–3.23	40	5	0.006	0.9	43.95	88.34	0.10
Ideal Solution						48.56	88.32	
Non-Ideal Solution						40.05	147.13	

#### 3.2.3 Results of the third scenario

HMOGWO is used for simultaneous EE maximization and GWP minimization in the last scenario. The mathematical representation of the cost functions and their corresponding decision variables’ limitations are presented in Section 3.1 and 2.6, correspondingly.

**Fig 9:**
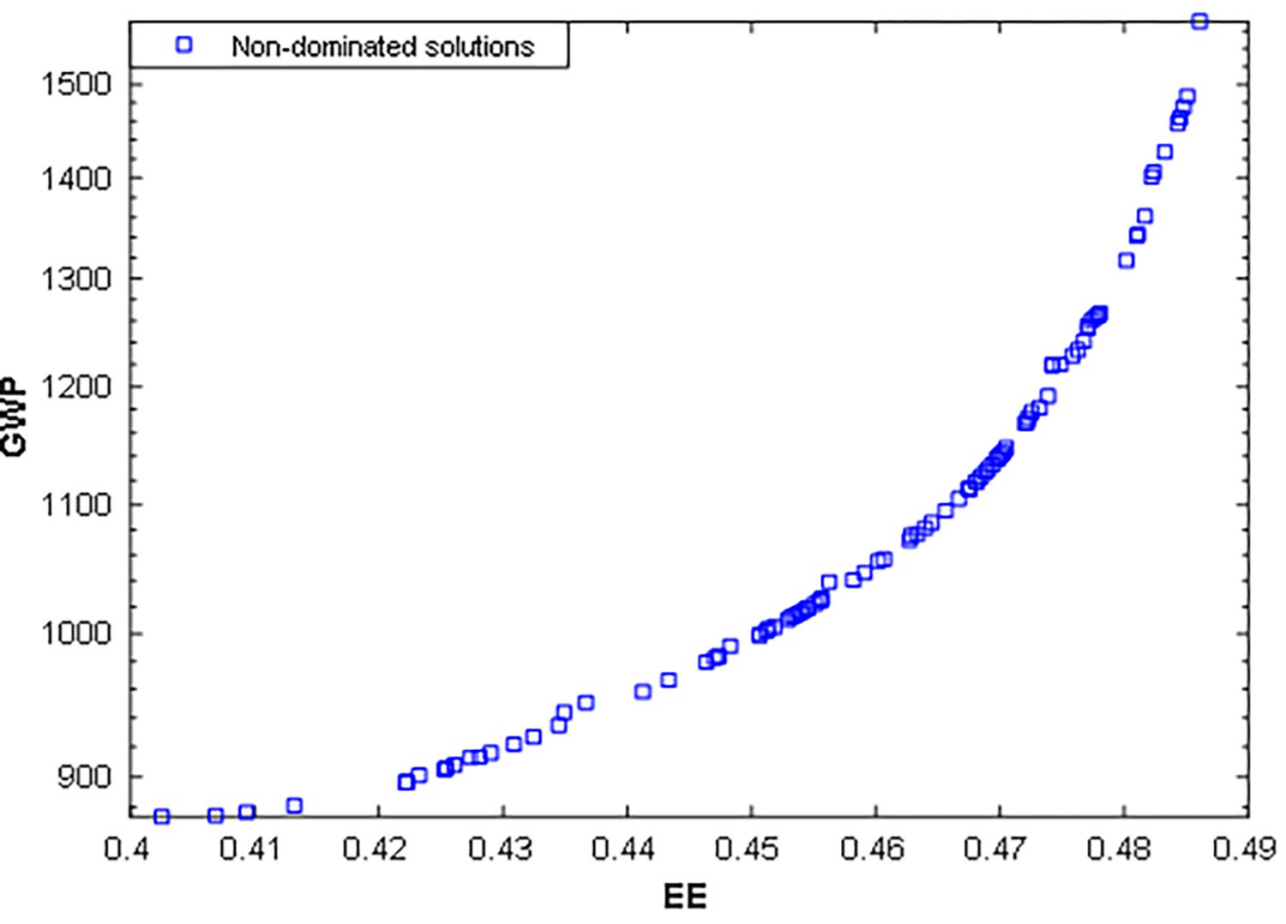
Pareto optimal solutions of the third scenario using HMOGWO.

Fig 9 illustrates the bi-objective optimization results of EE and GWP as a Pareto optimal front. The improvement of the EE from 0.40 to 0.49 also increases the GWP from 900 to 1572 kgCO_2_e per year. The best solutions recommended by the TOPSIS and LINMAP methods are also highlighted in Fig 9.

**Fig 10:**
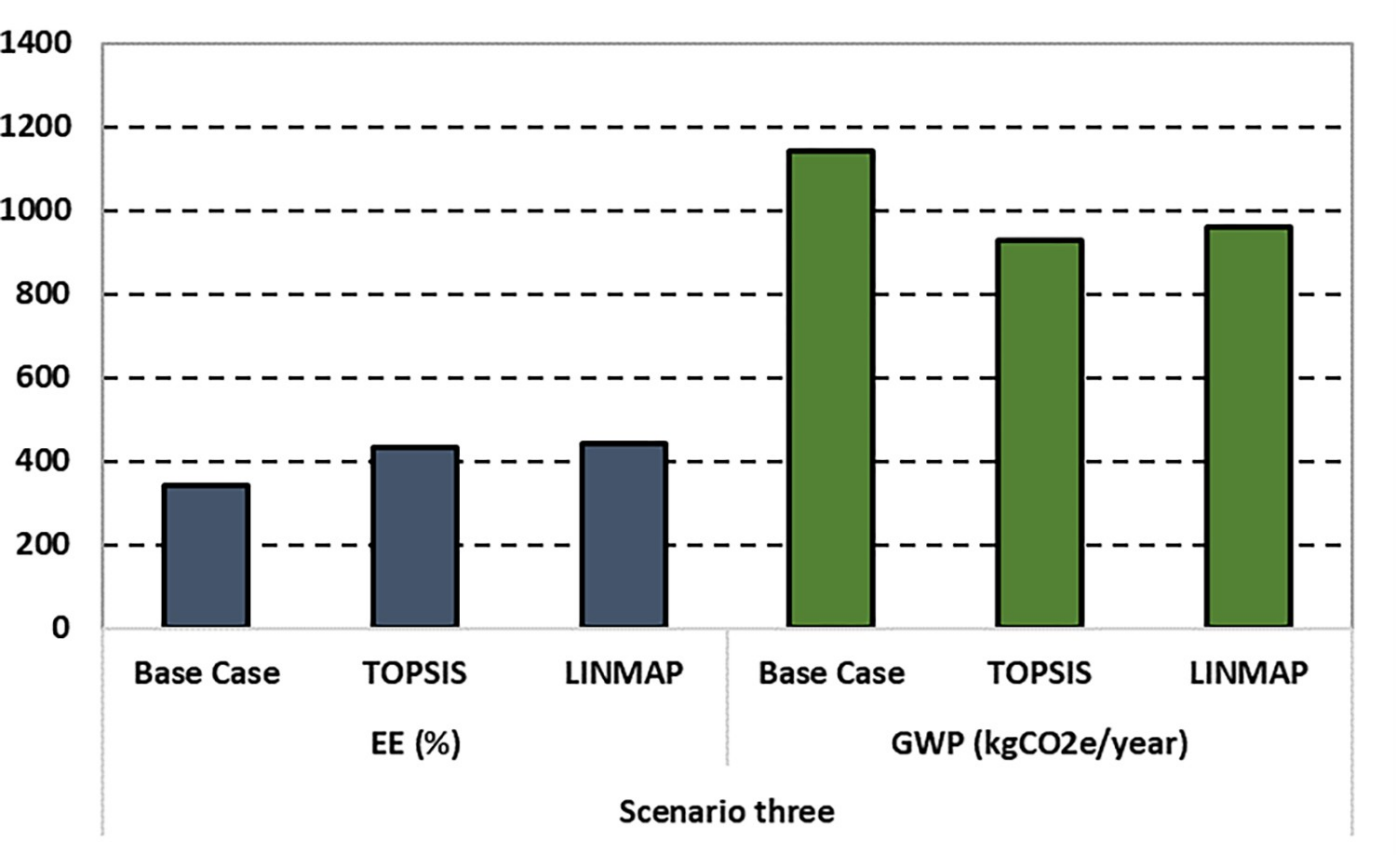
Comparison of EE and GWP for the third scenario.

The details of the recommended solutions set with their respective optimal design parameters are reported in Table 7. The results obtained by the TOPSIS technique are comparatively superior in terms of fitness values and their corresponding deviation index. Subsequently, Fig 10 compares the base case with the optimal solutions of the third case suggested by the TOPSIS and LINMAP decision-making methods. Please note that in Figs 6 and 10, the authors multiply the EE values by a factor of 10 to make the bar chart more visible and explicit to the readers.

**Table 7:** Optimal solutions obtained via decision-making methods for the third scenario.

Methods	Decision variables					Objectives		Deviation index
	T_evp_ [°C]	T_con_ [°C]	P_int_ [kPa]	$\dot{m}$ [kg s^–1^]	η_c_	EE [%]	GWP [kgCO_2_e per year]	
TOPSIS	–2.61	40	5	0.006	0.9	43.59	945.57	0.09
LINMAP	–3.02	40	5	0.006	0.9	43.78	958.16	0.11
Ideal Solution						48.61	874.71	
Non-Ideal Solution						40.25	1571.26	

The optimal solutions for all three cases indicate that a higher evaporator temperature, compressor efficiency, lower condenser temperature, intermediate stage pressure, and refrigerant mass flow rate are preferable for maximum EE and minimum CAPEX, OPEX, and GWP. The evaporator temperature changes slightly (–2.61 to –6.74°C) for all three optimization cases, but a higher value of evaporator temperature is generally noticeable for all cases. A higher evaporator temperature decreases the pressure ratio between the first and intermediate stages and the compression work required by the first stage compressor. This reduces the exergy destruction, energy consumption, OPEX, and GWP and increases EE. A lower evaporator temperature, by contrast, increases the temperature difference between the refrigerant and the heat source, hence reducing the heat transfer area and the CAPEX of the evaporator. However, it increases the system’s compression work, OPEX, and GWP and decreases the EE. This contradictory issue indicates a trade-off between CAPEX and other objectives such as OPEX and EE of the overall system. Therefore, the optimal evaporator temperature of the first case study, EE vs. CAPEX (–6.74°C), is comparatively higher than the second EE vs. OPEX (–2.93°C) and the third case study EE vs. GWP (–2.61°C).

On the other hand, a constant lower condenser temperature (40°C) is observed for all three optimum cases. Here, a lower condenser temperature reduces the second-stage compression pressure ratio, resulting in reduced power consumption, exergy destruction in the condenser, OPEX, GWP, and increased EE. Similarly, the algorithm suggests a lower intermediate stage pressure for all three optimum cases. Decreased intermediate stage pressure minimizes the first stage compression work, overall compression work, and OPEX. By contrast, the rise in intermediate stage pressure increases the pressure ratio of the first stage compression and increases the corresponding compression work, cost, and condenser cost. It has been seen from the optimal solutions for all three cases that a lower refrigerant mass flow rate is recommended. A lower mass flow rate decreases the system’s compression work and exergy destruction. Moreover, the algorithm suggests increased compressor efficiency (90%) for each optimization case. A more efficient compressor can convert the highest amount of provided electric energy to compression work, reducing exergy destruction and overall energy consumption of the system.

Moreover, all the four objectives (surrogate models) are further combined to run another simulation and see the difference between the optimal solutions of the first three bi-objective cases and the fourth objective case. It can be seen from the reported results in Table 8 that the bi-objective cases produce results slightly better than the four-objective cases. Since the trade-off among the objectives gets more complex in the case of four-objective optimization, the optimal solution must satisfy more criteria. The solutions are slightly worse compared to the previous bi-objective cases.

**Table 8:** Fourth objective optimization of the considered case.

Methods	Decision variables					Objectives				Deviation index
	T_evp_ [°C]	T_con_ [°C]	P_int_ [kPa]	m[kg s^–1^]	η_c_	EE [%]	CAPEX	OPEX	GWP	
							[USD/year]	[USD/year]	[KgCO2e/year]	
TOPSIS	-7.01	40.04	5.06	0.01	0.89	45.55	1346.42	98.28	1059.78	0.18
LINMAP	-1.53	40.03	5.06	0.01	0.89	43.12	1267.15	86.98	938.17	0.15
Ideal Solution						47.96	1241.24	83.29	898.48	
Non-Ideal Solution						40.96	1545.27	132.68	1430.43	

#### 3.2.5. Comparison of the optimization results

The best results accomplished for all three scenarios engaging HMOGWO are further compared with the best results achieved by MOGWO and NSGA-II in Table 10 (parameters of the algorithms are shown in Table 9). The parameters reported in Table 9 indicate that the proposed HMOGWO needs two extra parameters compared to the basic MOGWO algorithm, and it needs significantly fewer parameters than NSGA-II. Additionally, utilizing fewer parameters than the NSGA-II, it can obtain better optimal results within a short computation period.

**Table 9:** Parameter settings of the NSGA-II, MOGWO, and HMOGWO.

Parameter	Values		
	NSGA-II	MOGWO	HMOGWO
Iteration	250	100	100
Population type	Double Vector		
Population	100	100	100
Selection	Tournament	Roulette-wheel	Roulette-wheel
Crossover type	Intermediate		
Crossover percentage	70%		70%
Mutation	Constraint Dependent		
Random migrant	20%		
Migration	Forward		
Pareto fraction	0.6		
Archive size		100	100
F_max_ and F_min_			1.50 and 0.25
a		2 to 0	2 to 0

It is explicit that the results (Table 10) obtained by HMOGWO for the third scenario are comparable with NSGA-II and MOGWO. In contrast, the results of the HMOGWO for the first and second scenarios are better than NSGA-II and MOGWO. It is also noticeable from Table 10 that the HMOGWO can achieve superior results with fewer parameters than NSGA-II, making it computationally inexpensive and saving the time of trial and error-based parameter tuning. Among the three algorithms, the MOGWO works based on the position update mechanism of wolves following the best three wolves. NSGA-II works depending on the evolutionary crossover and mutation operator. By contrast, the proposed HMOGWO incorporates the evolutionary crossover and mutation operators of DE with the novel velocity and position update mechanism of MOGWO. Incorporating the features of the velocity and DE algorithm into the MOGWO algorithm increases the exploration ability of the proposed hybrid algorithm and provides superior solutions.

**Table 10:** Comparison of the optimum solutions obtained by MOGWO, NSGA-II, and HMOGWO.

Scenario	Index	Algorithms		
		MOGWO	NSGA-II	HMOGWO
**First**	EE [%]	45.24	45.32	45.70
	CAPEX [USD per year]	1305.05	1307.16	1304.97
**Second**	EE [%]	43.61	43.73	43.85
	OPEX [USD per year]	87.12	88.03	87.06
**Third**	EE [%]	43.18	43.09	43.59
	GWP [kgCO_2_e per year]	938.14	935.96	945.57
**Computation time**	seconds	135	190	147

The statistical analysis of the Pareto optimal solutions is done to compare the algorithms’ performances further. The statistical indexes are significant for comparing and measuring the algorithms’ superiority. Here, we considered five indexes for comparison: mean, median, minimum, maximum, and standard deviation (Std) values. We maximized the EE and minimized the CAPEX, OPEX, and GWP among the three optimization scenarios. For EE, the higher the value is, the better the solution, and for others, the lower value indicates better solutions. In Table 11, the best indexes for all three cases are made bold. For case 1, the HMOGWO provides the best values for a total of five indexes (2 for EE and 3 for CAPEX), whereas the MOGWO and NSGA-II provide the best values for two indexes, respectively. Similarly, for cases 2 and 3, the HMOGWO provides the best values for 4 and 7 indexes, while MOGWO provides the best values for 3 and 2 indexes, and NSGA-II obtains the best values for 3 and 0 indexes. These statistical comparisons also validate the superiority of the proposed novel HMOGWO algorithm.

**Table 11:** Statistical analysis and comparison of the optimum results obtained by three algorithms.

	NSGA-II	MOGWO	HMOGWO	NSGA-II	MOGWO	HMOGWO
	**EE**			**CAPEX**		
Mean	**0.466**	0.462	0.463	1410.705	1382.821	**1375.229**
Median	0.473	0.463	**0.474**	1412.000	**1341.050**	1346.363
Minimum	0.419	**0.422**	0.419	1220.600	1218.898	**1218.519**
Maximum	0.486	0.486	0.486	**1606.800**	1630.800	1621.081
Std	0.019	0.018	**0.016**	114.197	119.233	**101.289**
	**EE**			**OPEX**		
Mean	**0.463**	0.455	0.459	109.123	**100.619**	108.027
Median	**0.472**	0.458	0.469	108.560	**96.557**	105.336
Minimum	**0.404**	0.400	0.403	81.369	81.066	**81.021**
Maximum	0.485	0.486	**0.487**	139.000	146.230	**146.154**
Std	0.023	**0.022**	0.027	17.824	15.979	**14.192**
	**EE**			**GWP**		
Mean	0.457	0.458	**0.459**	1113.097	1103.134	**1101.574**
Median	0.463	0.463	**0.469**	1077.850	1075.850	**1068.237**
Minimum	0.402	0.403	**0.404**	874.590	873.730	**872.198**
Maximum	0.484	0.485	**0.486**	1438.900	1571.300	1569.024
Std	0.023	**0.021**	0.026	171.857	**166.069**	210.899

### 3.3 Pareto characterization

Generally, in MOO problems, we get a set of non-dominated solutions called the Pareto optimal front. It is common to obtain a single point from the non-dominated solution sets by utilizing higher-order information and abandoning the remaining solutions [57]. This procedure is somehow influential. However, it cannot offer detailed information about the solution set. By contrast, data analytical methods, also known as Pareto characterization, can help us determine the hidden pattern and information in the solution sets [58]. Therefore, the Pareto optimal solutions are clustered using the K-means technique to get credible information about the decision variables, which could be helpful to the industrialist to operate the plant in optimal condition successfully.

**Fig 11:**
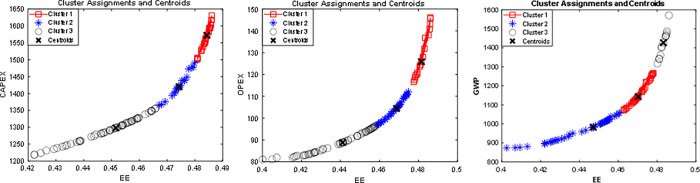
K-means clustering of the Pareto optimal solutions, a) CAPEX, b) OPEX, and c) GWP.

The Pareto optimal data sets of each Pareto frontier are divided into three different clusters, such that each cluster represents different operating conditions with different sets of objective function values. The cluster sets of Pareto optimal solutions in the objective spaces are presented in Fig 11. The 100 optimum solutions are split into three groups, labeled cluster #1, cluster #2, and cluster #3, to illustrate if the design variable sets of the points within each cluster are comparable. A centroid point is displayed in each cluster, which may be regarded as the best optimum solution among the other solutions in the cluster. Additionally, each cluster’s respective decision variable sets (evaporator temperature, condenser temperature, intermediate stage pressure, refrigerant mass flow rate, and compressor efficiency) are presented in S2 File.

## 4. Conclusions

The refrigeration system’s non-linear, non-convex, and multi-modal nature makes optimization challenging. Additionally, it imposes an additional computational burden on obtaining a feasible solution for multi-objective optimization with many conflicting objectives. To tackle these issues, this article proposes a novel approach for modeling and bi-objective optimization of a two-stage vapor compression refrigeration system, considering both surrogate modeling and an HMOGWO technique for the first time.

The overall system is designed using rigorous process simulation software Aspen Hysys and modeling of the system is done on Design Expert software. RSM investigates the impacts of each design variable (evaporator and condenser temperature, intermediate stage pressure, refrigerant mass flow rate through the evaporator, and compressor efficiency) on the overall system where the individual and pairwise (interaction and self-interaction) impacts are comprehensively investigated. Four surrogate models (EE, CAPEX, OPEX, GWP) have been developed based on the system’s input and output data sets. Three bi-objective scenarios requiring a different difficulty level are optimized using the HMOGWO algorithm. Two robust and efficient decision-making methods were further employed to determine the optimal solution from Pareto optimal solution sets. Finally, the K-means clustering method is applied for Pareto characterization.

Based on the fitted correlation, the RSM recommended the quadratic models as optimal for all four considered cases (EE, CAPEX, OPEX, and GWP). The fit statistics and the model summaries show that all the quadratic polynomial models are highly significant, with a confidence level of 95%. Moreover, the coefficient of determination values (R^2^), adjusted R^2,^ and predicted R^2^ values are more than 0.994 for all the models, indicating higher accuracy of the built models. The ANOVA test results indicate that the condenser temperature is EE’s most dominant decision variable, with a 52.74% contribution. In contrast, the evaporator temperature has the highest impact on the other three models, with 74.3%, 75.2%, and 75.2% contributions in CAPEX, OPEX, and GWP.

The optimal solutions are compared with the base case. It has been noticed that the HMOGWO successfully optimized all the considered cases with a minimum amount of computational effort and deviation index. In the first scenario, EE and CAPEX are optimized by 33.41% and 7.45%, respectively. Consequently, EE and OPEX are improved for the second scenario, up to 27.44% and 19.00%. Likewise, EE and GWP are optimized by 27.18% and 19.10% for the last scenario, respectively. Furthermore, the optimum design variables and objectives reveal that a greater evaporator temperature and compressor efficiency and a lower condenser temperature, intermediate stage pressure, and refrigerant mass flow rate are essential for the overall system’s optimal operation, cost and GWP.

Finally, the optimal results of HMOGWO are further compared with MOGWO and NSGA-II, where the first method outperformed the others and accomplished superior results for two scenarios. Moreover, it has been noticed that the proposed algorithm requires significantly less parameter handling, which makes it computationally inexpensive and easily adaptable for optimization. Furthermore, Pareto characterization is done using the K-means clustering technique to understand the hidden pattern of the cost functions and decision variables, which could be helpful to researchers and industrialists. Finally, it can be concluded that the modeling and optimization techniques presented in this research can be easily adapted to any refrigeration system.

## Supporting information

S1 File The details of the 4E analysis, response surface method (RSM), and multi-objective optimization. (DOCX)

S2 File Data. (XLSX)

S3 File Pareto characterization for the decision variables. (DOCX)

## Abbrevation

A: heat transfer area [m^2^]
$\vec{a}$: acceleration constant
$\vec{A}$: coefficient vector
C: capital cost [USD]
$\vec{C}$: coefficient vector
$C_{co_{2}}$: cost of avoided CO_2_ [USD kgCO_2_e^–1^]
Cl_i_: solution recommended by the TOPSIS method
COP: coefficient of performance
$\vec{D}$: distance vector
df: degrees of freedom
E_total_: total electricity consumption [kWh]
Edi+: Euclidean distance from the ideal solution
E_di-_: Euclidean distance from the non-ideal solution
E_x_, E_eff_: exergy efficiency
F: scaling factor
F value: Fisher’s statistical test value
Fijn: non-dimensionalized matrix of the Pareto solutions
h: specific enthalpy [kJ kg^–1^]
$\dot{m}$: mass flow rate [kg s^–1^]
${\dot{m}}_{co_{2}}$: yearly greenhouse gas emission [kgCO_2_e]
N: annual operation time [hours]
P: pressure [kPa]
P value: probability value
$\dot{Q}$: heat [kW]
r: mass flow ratio
$\vec{r}$: random vector
R^2^: coefficient of determination
S: specific entropy [kJ kg^–1^ K^–1^]
T: temperature [K]
U: heat transfer coefficient [kW m^–2^ K^–1^]
$\dot{W}$: work [kW]
$\vec{X}$: position vector
X: exergy rate [kW]
X_d_: exergy destruction [kW]
Z: capital investment and maintenance cost rate [USD year^–1^]
Greek letters *η*: efficiency [%]
η_el_: electrical efficiency [%]
η_me_: mechanical efficiency [%]
η_c_: total efficiency [%]
α, β, γ: position of the best three wolves
$\mu_{{co}_{2}}$: CO_2_e emission factor [kg kWh^–1^]
φ: maintenance factor
Subscript 1, 2 … a, b, c…: number of states
ac: actual
cap: capita
capex: capital expenditure
comp: compressor, component
con: condenser
des: destruction, destroyed
el: electrical
env: environmental
evp: evaporator
exp: expansion valve
fc: flash chamber
H: high pressure stage
HTV: high pressure stage thermal expansion valve
id+: ideal
id-: non-ideal
int: intermediate stage
is: isentropic
L: low pressure stage
LTV: low pressure stage thermal expansion valve
me: mechanica
Mx it: maximum iteration
o: dead states
opex: operational expenditure
r: refrigerant
split: splitting
T: total
Abreviatures 4E: energy-exergy, economic and environmental
ANN: artificial neural networks
ANOVA: analysis of variance
BBD: box-behnken design
CAPEX: capital expenditure
CCD: central composite design
CCRD: central composite rotational design
CRF: capital recovery factor
CV: coefficient of variation
DE: differential evolution
DOE: design of experiment
EE: exergetic efficiency
GA: genetic algorithm
GWO: grey wolf optimizer
GWP: global warming potential
HFC: hydrofluorocarbon
HTC: heat transfer coefficient
IPCC: Intergovernmental Panel on Climate Change
LINMAP: linear programming technique for multi-dimensional analysis of preference
LMTD: logarithmic mean temperature difference
MOGA: multi objective genetic algorithm
MOGWO: multi-objective grey wolf optimizer
MOO: multi-objective optimization
MVC: mechanical vapor compression system
NLP: non-linear programming
NSGA II: non-dominated sorting genetic algorithm II
ODP: ozone depletion potential
OPEX: operational expenditure
ORC: organic Rankine cycle
PA: Pareto archive
P-h: pressure-enthalpy
PSO: particle swarm optimization
RSM: response surface methods
SVM: support vector machines
STD: standard deviation
TOPSIS: technique for order of preference by similarity to the ideal solution
VCRS: vapor compression refrigeration system
