# Supplementary material for: 4E analysis of a two-stage refrigeration system through surrogate models based on response surface methods and hybrid grey wolf optimizer
Source: PLoS One. 2023 Feb 3;18(2):e0272160. doi: 10.1371/journal.pone.0272160 (PMC9897521; doi:10.1371/journal.pone.0272160)
Supplement: S1 File — (DOCX) [file pone.0272160.s001.docx]

### S1 File:

### 2.3.1 Energy analysis

This section illustrates the necessary equations to perform the energetic analysis of the overall system. Hence, the mass balance of the total system, Eq. (1).

| $\Sigma\dot{m}=0$ | (1) |
| --- | --- |

The energy balance of the system, Eq. (2).

| $\Sigma\dot{Q}+ \Sigma\dot{W}+ \Sigma\dot{m}h=0$ | (2) |
| --- | --- |

Exergy balance of the system, Eq. (3).

| $\Sigma X_{in}-\Sigma X_{out}-\Sigma X_{heat}-\Sigma\left( X-X_{destroyed} \right)_{work}=0$ | (3) |
| --- | --- |

Cooling capacity at the evaporator, Eq. (4).

| $\dot{Q}_{evp}=\dot{m}_{revp}(h_{a}-h_{h})$ | (4) |
| --- | --- |

The refrigerant mass flow rate through the condenser is the sum of the mass flow rate through the evaporator and flash chamber. Here, $\dot{m}_{r,con}$, $\dot{m}_{revp}$, and$\dot{m}_{rfc}$ are the mass flow through condenser, evaporator, and flash chamber, Eq. (5).

| $\dot{m}_{r,con}=\dot{m}_{revp}+\dot{m}_{rfc}$ | (5) |
| --- | --- |

The amount of work performed by the low-pressure stage compressor, Eq. (6).

| $\dot{W}_{1}=\dot{m}_{revp}(h_{b2}-h_{a})$ | (6) |
| --- | --- |

Similarly, high-pressure stage compressor work can be calculated by Eq. (7).

| $\dot{W}_{2}=\dot{m}_{rcon}(h_{d2}-h_{c})$ | (7) |
| --- | --- |

The isentropic efficiency of the low and high stage compressor can be defined as Eq. (8), being correlated with the pressure ratio, Eq. (9) [7].

| $\eta_{is}=\eta_{is1}=\eta_{is2}=\frac{h_{b}-h_{a}}{h_{b2}-h_{a}}= \frac{h_{d}-h_{c}}{h_{d2}-h_{c}}$ | (8) |
| --- | --- |

| $\eta_{is}=\left( 0.85- 0.046667 \right)\frac{p_{out}}{p_{in}}$ | (9) |
| --- | --- |

Actual work performed by the first compressor can be written as Eq. (10) [7].

| $\dot{W}_{L}=\frac{\dot{W}_{1}}{\eta_{el} \eta_{me}}$=$\frac{\dot{W}_{1}}{\eta_{c}}$ | (10) |
| --- | --- |

$\dot{W}_{L}, \dot{W}_{H}$ are the actual work performed by the first compressor and second compressor, $\eta_{el}$ means electric efficiency of the compressors, $\eta_{me}$ indicates the mechanical efficiency of the compressors, $\eta_{c}$ represents the total efficiency of the compressors and is the product of both efficiencies. Similarly, the second compressor's amount of work that has to be done can be written as Eq. (11).

| $\dot{W}_{H}=\frac{\dot{W}_{2}}{\eta_{el} \eta_{me}}$ = $\frac{\dot{W}_{2}}{\eta_{c}}$ | (11) |
| --- | --- |

The total amount of electricity absorbed by the two compressors, Eq. (12).

| $\dot{W}_{T}= \dot{W}_{L}+ \dot{W}_{H}$ | (12) |
| --- | --- |

Heat transfer through the condenser can be defined by Eq. (13).

| $\dot{Q}_{con}=\dot{m}_{rcon}(h_{d2}-h_{e})$ | (13) |
| --- | --- |

$h_{d2}$ and $h_{e}$ refer to the inlet and outlet condition of the condenser. The coefficient of performance (COP) is the ratio of cooling capacity to the compressors electricity consumption, and it can be defined as Eq. (14).

| $COP= \frac{\dot{Q}_{evp}}{\dot{W}_{T}}$ | (14) |
| --- | --- |

### 2.3.2 Exergy analysis

Exergy analysis is proposed to measure the exergy destruction at each component and the overall exergetic efficiency of the system. Exergy destruction by the low and high stage compressors, evaporator, condenser, flash tank, and expansion valves can be expressed as Eq. (15) to (21).

| ${Xd}_{Lcomp}=\dot{m}_{revp} \left[ \left( h_{a}-h_{b2} \right)-T_{0}\left( S_{a}-S_{b2} \right) \right]+\dot{W}_{L}$ | (15) |
| --- | --- |

| ${Xd}_{Hcomp}=\dot{m}_{rcon}\left[ \left( h_{c}-h_{d2} \right)-T_{0}\left( S_{c}-S_{d2} \right) \right]+\dot{W}_{H}$ | (16) |
| --- | --- |

| ${Xd}_{evp}=\dot{m}_{revp}\left[ \left( h_{h}-h_{a} \right)-T_{0}\left( S_{h}-S_{a} \right) \right]+\dot{Q}_{evp}(1-\frac{T_{0}}{T_{L}})$ | (17) |
| --- | --- |

| ${Xd}_{cond}=\dot{m}_{rcon}\left[ \left( h_{d2}-h_{e} \right)-T_{0}\left( S_{d2}-S_{e} \right) \right]+\dot{Q}_{con}(1-\frac{T_{0}}{T_{H}})$ | (18) |
| --- | --- |

| ${Xd}_{flash}=\dot{m}_{revp}\left[ \left( h_{b2}+h_{e}-h_{g}-h_{f} \right)-T_{0} \left( S_{b2}+S_{e}-S_{g}-S_{f} \right) \right]+\dot{m}_{rcon}[\left( h_{f}-h_{c} \right)-T_{0}(S_{f}-S_{c})]$ | (19) |
| --- | --- |

| $Xd_{HTV}=\dot{m}_{rsplit}T_{0}(S_{e}-S_{f})$ | (20) |
| --- | --- |

| $Xd_{LTV}=\dot{m}_{revp}T_{0}(S_{g}-S_{h})$ | (21) |
| --- | --- |

The total exergy destruction is the summation of the exergy destruction by the first and second stage compressors, evaporator, condenser, flash tank, and expansion valves as shown in Eq. (22), and the exergy efficiency of the overall system can be represented by Eq. (23).

| $Xd_{T}= Xd_{Lcomp}+ {Xd}_{Hcomp}+ {Xd}_{evp}+ {Xd}_{con}+ {Xd}_{flash}+ Xd_{HTV}+ Xd_{LTV}$ | (22) |
| --- | --- |

| $E_{x} E_{eff}= \frac{\dot{W}_{T}-Xd_{T}}{\dot{W}_{T}}$ | (23) |
| --- | --- |

### 2.3.3 Economic analysis

In economic analysis, we consider the capital and maintenance cost (capital expenditure, CAPEX), operation cost (OPEX), and environmental cost (due to greenhouse gas emissions) of the total system. The total cost of the system can be expressed as Eq. (24).

| $Z_{total}=Z_{env}+ Z_{OPEX}+\Sigma Z_{CAPEX}$ | (24) |
| --- | --- |

The capital cost of any component can be expressed by Eq. (25) [9].

| $Z_{CAPEX}=C_{k}$φ$CRF$ | (25) |
| --- | --- |

C_k_ is the capital cost of the component, φ represents the maintenance factor, and CRF represents the capital recovery factor, which can be calculated as Eq. (26) [42].

| $CRF= \frac{i\left( i+1 \right)^{n}}{\left( i+1 \right)^{n}-1}$ | (26) |
| --- | --- |

The capital cost of the components such as high and low stage pressure compressor, evaporator, condenser, flash tank, high and low stage pressure thermal expansion valves can be expressed by Eq. (27) to (33) [9,41,43].

| $C_{Hcomp}=9624.2 \dot{W}_{H}^{0.46}$ | (27) |
| --- | --- |

| $C_{Lcomp}=10167.5 \dot{W}_{L}^{0.46}$ | (28) |
| --- | --- |

| $C_{evp}=1397 A_{evp}^{0.89}$ | (29) |
| --- | --- |

| $C_{cond}=1397 A_{con}^{0.89}$ | (30) |
| --- | --- |

| $C_{flash}=280.3 \dot{m}_{flash}$ | (31) |
| --- | --- |

| $C_{HTV}=114.5 \dot{m}_{HP}$ | (32) |
| --- | --- |

| $C_{LTV}=114.5 \dot{m}_{LP}$ | (33) |
| --- | --- |

The overall system's total capital and maintenance cost rate can be expressed as the summation of all components capital and maintenance cost, Eq. (34).

| $Z_{CAPEX}= Z_{Hcomp}+Z_{Lcomp}+ Z_{eva}+ Z_{con}+ Z_{flash}+ Z_{HTV}+ Z_{LTV}$ | (34) |
| --- | --- |

The operational cost rate of the system can be represented as Eq. (35) [9,41].

| $Z_{OPEX}=N\dot{W}_{total} C_{elc}$ | (35) |
| --- | --- |

### 2.3.4 Environmental analysis

Since greenhouse gas emissions are increasing rapidly in the environment, which is the main culprit of global warming, it is essential to consider thermal systems modeling. In this research, we are considering the total amount of CO_2_e emission as global warming potential. The total cost can be calculated as Eq. (36) [9,43]~~.~~

| $Z_{env}=\dot{m}_{co_{2}}C_{co_{2}}$ | (36) |
| --- | --- |

$Z_{env}$ is the total cost of avoided CO_2_e and $\dot{m}_{co_{2}}$ is the yearly greenhouse gas emission, which can be expressed by Eq. (37) [41,43].

| $\dot{m}_{co_{2}}=\mu_{{co}_{2}} \dot{E}_{total}$ | (37) |
| --- | --- |

$E_{total}$is the total electricity consumption and $\mu_{{co}_{2}}$ is the CO_2_e emission factor. Values are listed in Table 1, and other parameters are calculated during the process analysis.

## 2.4 Response Surface Method (RSM)


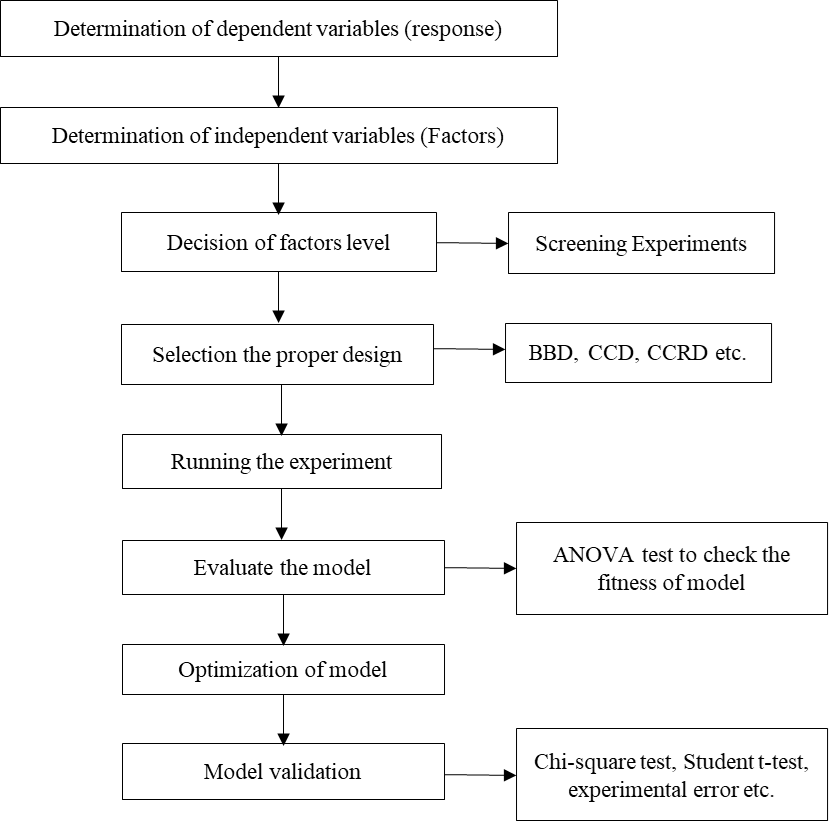


S1 Fig 1. Main steps of the Response Surface Method.

## 2.5 Multi-objective optimization

Highly efficient thermal systems require more capital, maintenance, and operational costs. To minimize the cost, some amount of efficiency needs to be sacrificed. This indicates the conflicting nature of the objectives [46,47]. Moreover, optimizing these systems by single objective optimization or by deterministic techniques is not sufficient to address the issues completely. By contrast, MOO is performed to achieve maximum thermal efficiency simultaneously, and system stability at a minimum required cost. The MOO optimizes several conflicting objectives concurrently without violating system constraints [48].

In this research, the authors performed thermo-economic and thermo-environmental optimization of a two-stage VCRS. In thermo-economic and thermo-environmental formulations, the laws of thermodynamics and environmental constraints are satisfied [49] while optimizing for maximum EE, minimum CAPEX, OPEX, and GWP. In MOO, numerous optimal solutions satisfy the conflicting objectives up to a certain level. A gain in one objective is a loss of another objective. The achieved solutions spread through the search space where others dominate some solutions, and each best solution will dominate the nearby worst solutions. The sorted non-dominated solutions are presented as a Pareto optimal front [50]. Figure 2 represents the general structure of a Pareto optimal frontier for two minimization objectives.

| 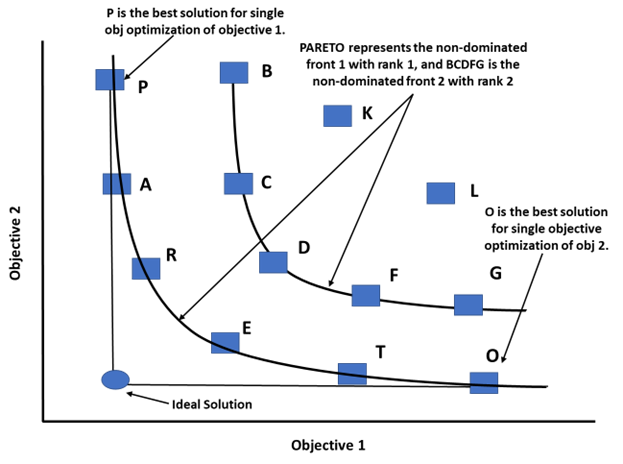 |
| --- |

S1 Fig 2. ****A generalized structure of Pareto optimal front*****.*

S1 Table 1. ANOVA analysis for modeling of the EE of the designed two-stage VCRS.

| **Source** | **sum of squares** | **df** | **mean square** | **F-value** | **P-value** | **Significant** | **Contribution** |
| --- | --- | --- | --- | --- | --- | --- | --- |
| Model | 0.066 | 20 | 0.003 | 108.320 | < 0.0001 | yes | 99.40 |
| A-Evp Temp | 0.008 | 1 | 0.008 | 267.210 | < 0.0001 | yes | 12.27 |
| B-Cond Temp | 0.036 | 1 | 0.036 | 1169.990 | < 0.0001 | yes | 52.74 |
| C-Int Pres | 0.008 | 1 | 0.008 | 249.270 | < 0.0001 | yes | 11.37 |
| D-Mass Flow | 0.000 | 1 | 0.000 | 0.101 | 0.756 | no | 0.01 |
| E-Efficiency | 0.006 | 1 | 0.006 | 208.400 | < 0.0001 | yes | 9.58 |
| AB | 0.000 | 1 | 0.000 | 0.211 | 0.655 | no | 0.01 |
| AC | 0.000 | 1 | 0.000 | 0.999 | 0.339 | no | 0 |
| AD | 0.000 | 1 | 0.000 | 0.769 | 0.399 | no | 0 |
| AE | 0.000 | 1 | 0.000 | 3.990 | 0.071 | no | 0.15 |
| BC | 0.000 | 1 | 0.000 | 9.280 | 0.011 | yes | 0.45 |
| BD | 0.000 | 1 | 0.000 | 0.005 | 0.947 | no | 0 |
| BE | 0.000 | 1 | 0.000 | 0.680 | 0.427 | no | 0 |
| CD | 0.000 | 1 | 0.000 | 0.001 | 0.980 | no | 0 |
| CE | 0.000 | 1 | 0.000 | 0.079 | 0.784 | no | 0.01 |
| DE | 0.000 | 1 | 0.000 | 2.280 | 0.159 | no | 0.15 |
| A² | 0.007 | 1 | 0.007 | 220.290 | < 0.0001 | yes | 10.18 |
| B² | 0.000 | 1 | 0.000 | 10.760 | 0.007 | yes | 0.45 |
| C² | 0.001 | 1 | 0.001 | 16.540 | 0.002 | yes | 0.75 |
| D² | 0.000 | 1 | 0.000 | 0.052 | 0.824 | no | 0.01 |
| E² | 0.000 | 1 | 0.000 | 0.004 | 0.950 | no | 0 |

Note that in the four regression models of EE, CAPEX, OPEX, and GWP, the term x_1_ is A (evaporator temperature), x_2_ is B (condenser temperature), x_3_ is C (intermediate stage pressure), x_4_ is D (refrigerant mass flow rate), and x_5_ is E (compressor's efficiency).

The corresponding regression model of EE is presented in Eq. (38).

| $EE=1.30505 - 0.00196x_{1} - 0.02269x_{2}-0.01822x_{3}-54.4702x_{4}- 0.17534 x_{5}+ 0.000013x_{1}x_{2} -0.00014x_{1}x_{3} + 0.242762x_{1}x_{4} -0.00553x_{1}x_{5}+ 0.000844x_{2}x_{3}+ 0.037479x_{2}x_{4} -0.00457x_{2}x_{5} + 0.069825x_{3}x_{4} + 0.007771x_{3}x_{5}+ 83.69256x_{4}x_{5} -0.00015 x_{1}^{2} + 0.000134 x_{2}^{2}-0.00416x_{3}^{2} -933.441x_{4}^{2}+0.026413x_{5}^{2}$ | (38) |
| --- | --- |

S1 Table 2. ANOVA analysis for modeling of the CAPEX of the designed two-stage VCRS.

| **Source** | **sum of squares** | **df** | **mean square** | **F-value** | **P-value** | **Significant** | **Contribution** |
| --- | --- | --- | --- | --- | --- | --- | --- |
| Model | 631300 | 20 | 31566.950 | 254.560 | < 0.0001 | yes | 100 |
| A-Evp Temp | 469100 | 1 | 469100 | 3782.590 | < 0.0001 | yes | 74.3 |
| B-Cond Temp | 28993.580 | 1 | 28993.580 | 233.800 | < 0.0001 | yes | 4.6 |
| C-Int Pres | 31502.740 | 1 | 31502.740 | 254.040 | < 0.0001 | yes | 5 |
| D-Mass Flow | 68336.260 | 1 | 68336.260 | 551.070 | < 0.0001 | yes | 10.8 |
| E-Efficiency | 26557.900 | 1 | 26557.900 | 214.160 | < 0.0001 | yes | 4.2 |
| AB | 4.910 | 1 | 4.910 | 0.040 | 0.846 | no | 0 |
| AC | 1501.800 | 1 | 1501.800 | 12.110 | 0.005 | yes | 0.2 |
| AD | 682.980 | 1 | 682.980 | 5.510 | 0.039 | no | 0.1 |
| AE | 388.640 | 1 | 388.640 | 3.130 | 0.104 | no | 0.1 |
| BC | 13.690 | 1 | 13.690 | 0.110 | 0.746 | no | 0 |
| BD | 4.070 | 1 | 4.070 | 0.033 | 0.860 | no | 0 |
| BE | 100.150 | 1 | 100.150 | 0.808 | 0.388 | no | 0 |
| CD | 41.720 | 1 | 41.720 | 0.336 | 0.574 | no | 0 |
| CE | 161.360 | 1 | 161.360 | 1.300 | 0.278 | no | 0 |
| DE | 264.550 | 1 | 264.550 | 2.130 | 0.172 | no | 0 |
| A² | 79.790 | 1 | 79.790 | 0.643 | 0.440 | no | 0 |
| B² | 23.690 | 1 | 23.690 | 0.191 | 0.671 | no | 0 |
| C² | 3133.480 | 1 | 3133.480 | 25.270 | 0.000 | no | 0.5 |
| D² | 139.260 | 1 | 139.260 | 1.120 | 0.312 | no | 0 |
| E² | 361.570 | 1 | 361.570 | 2.920 | 0.116 | no | 0.1 |

The corresponding regression model of CAPEX, as Eq. (39).

| $CAPEX=742.65738 -{18.30764}_{x1}+ 18.14004x_{2} -40.65394x_{3}+72297.42111x_{4} -793.67923x_{5} -0.011082x_{1}x_{2}+ 0.968825x_{1}x_{3}-1306.69337x_{1}x_{4}+ 9.85701x_{1}x_{5} -0.185004x_{2}x_{3}+ 201.67032x_{2}x_{4}-10.00764x_{2}x_{5}+ 3229.60896x_{3}x_{4} -63.51408x_{3}x_{5}-{1.63E}^{05}x_{4}x_{5}-0.016493x_{1}^{2} -0.03595x_{2}^{2}+10.33554x_{3}^{2}+ {8.72E}^{06}x_{4}^{2}+1404.34685x_{5}^{2}$ | (39) |
| --- | --- |

S1 Table 3. ANOVA analysis for modeling of the OPEX of the designed two-stage VCRS.

| **Source** | **sum of squares** | **df** | **mean square** | **F-value** | **P-value** | **Significant** | **Contribution** |
| --- | --- | --- | --- | --- | --- | --- | --- |
| Model | 28090.533 | 20 | 1404.527 | 358.908 | 0.000 | yes | 100 |
| A-Evp Temp | 21121.381 | 1 | 21121.381 | 5397.288 | 0.000 | yes | 75.2 |
| B-Cond Temp | 1608.852 | 1 | 1608.852 | 411.121 | 0.000 | yes | 5.7 |
| C-Int Pres | 1222.854 | 1 | 1222.854 | 312.484 | 0.000 | yes | 4.4 |
| D-Mass Flow | 2314.031 | 1 | 2314.031 | 591.320 | 0.000 | yes | 8.2 |
| E-Efficiency | 1204.289 | 1 | 1204.289 | 307.740 | 0.000 | yes | 4.3 |
| AB | 1.197 | 1 | 1.197 | 0.306 | 0.591 | no | 0 |
| AC | 3.910 | 1 | 3.910 | 0.999 | 0.339 | no | 0 |
| AD | 100.802 | 1 | 100.802 | 25.759 | 0.000 | yes | 0.4 |
| AE | 60.447 | 1 | 60.447 | 15.446 | 0.002 | yes | 0.2 |
| BC | 2.073 | 1 | 2.073 | 0.530 | 0.482 | no | 0 |
| BD | 4.874 | 1 | 4.874 | 1.245 | 0.288 | no | 0.0 |
| BE | 8.627 | 1 | 8.627 | 2.204 | 0.166 | no | 0.0 |
| CD | 6.519 | 1 | 6.519 | 1.666 | 0.223 | no | 0.0 |
| CE | 15.791 | 1 | 15.791 | 4.035 | 0.070 | no | 0.1 |
| DE | 20.007 | 1 | 20.007 | 5.112 | 0.045 | no | 0.1 |
| A² | 203.433 | 1 | 203.433 | 51.985 | 0.000 | yes | 0.7 |
| B² | 0.002 | 1 | 0.002 | 0.001 | 0.982 | no | 0 |
| C² | 214.707 | 1 | 214.707 | 54.866 | 0.000 | yes | 0.8 |
| D² | 8.739 | 1 | 8.739 | 2.233 | 0.163 | no | 0 |
| E² | 15.349 | 1 | 15.349 | 3.922 | 0.073 | no | 0.1 |

The resultant regression model of OPEX is shown below, Eq. (40).

| $OPEX=7.85303 - 1.46563x_{1} + 2.78592x_{2}-15.26351x_{3} + 1138.73114x_{4}+ 13.41172x_{5}-0.005471x_{1}x_{2}-0.049432x_{1}x_{3}-502.00155x_{1}x_{4}+3.88739x_{1}x_{5} -0.071989x_{2}x_{3} + 220.7655x_{2}x_{4}-2.93714x_{2}x_{5}+1276.6005x_{3}x_{4}-19.8689x_{3}x_{5}-44729.01x_{4}x_{5}+0.026335x_{1}^{2}+0.000332x_{2}^{2}+2.70547x_{3}^{2}+2.18E^{06}x_{4}^{2}+289.35115x_{5}^{2}$ | (40) |
| --- | --- |

S1 Table 4. ANOVA analysis for modeling of the GWP of the designed two-stage VCRS.

| **Source** | **sum of squares** | **df** | **mean square** | **F-value** | **P-value** | **Significant** | **Contribution** |
| --- | --- | --- | --- | --- | --- | --- | --- |
| Model | 3250000 | 20 | 162500 | 358.910 | < 0.0001 | yes | 100 |
| A-Evp Temp | 2443000 | 1 | 2443000 | 5397.290 | < 0.0001 | yes | 75.2 |
| B-Cond Temp | 186100 | 1 | 186100 | 411.120 | < 0.0001 | yes | 5.7 |
| C-Int Pres | 141500 | 1 | 141500 | 312.480 | < 0.0001 | yes | 4.4 |
| D-Mass Flow | 267700 | 1 | 267700 | 591.320 | < 0.0001 | yes | 8.2 |
| E-Efficiency | 139300 | 1 | 139300 | 307.740 | < 0.0001 | yes | 4.3 |
| AB | 138.510 | 1 | 138.510 | 0.306 | 0.591 | no | 0 |
| AC | 452.280 | 1 | 452.280 | 0.999 | 0.339 | no | 0 |
| AD | 11661 | 1 | 11661 | 25.760 | 0.000 | yes | 0.4 |
| AE | 6992.660 | 1 | 6992.660 | 15.450 | 0.002 | yes | 0.2 |
| BC | 239.800 | 1 | 239.800 | 0.530 | 0.482 | no | 0 |
| BD | 563.800 | 1 | 563.800 | 1.250 | 0.288 | no | 0 |
| BE | 997.960 | 1 | 997.960 | 2.200 | 0.166 | no | 0 |
| CD | 754.110 | 1 | 754.110 | 1.670 | 0.223 | no | 0 |
| CE | 1826.720 | 1 | 1826.720 | 4.040 | 0.070 | no | 0.1 |
| DE | 2314.430 | 1 | 2314.430 | 5.110 | 0.045 | no | 0.1 |
| A² | 23533.520 | 1 | 23533.520 | 51.980 | < 0.0001 | yes | 0.7 |
| B² | 0.233 | 1 | 0.233 | 0.001 | 0.982 | no | 0 |
| C² | 24837.730 | 1 | 24837.730 | 54.870 | < 0.0001 | yes | 0.8 |
| D² | 1010.890 | 1 | 1010.890 | 2.230 | 0.163 | no | 0 |
| E² | 1775.650 | 1 | 1775.650 | 3.920 | 0.073 | no | 0.1 |

The regression model of GWP can be written as Eq. (41).

| $GWP= 84.46367-15.76369x_{1}+ 29.96413x_{2}-164.16748x_{3}+12247.686x_{4}+144.25052x_{5} -0.058845x_{1}x_{2}-0.531672x_{1}x_{3}-5399.30556x_{1}x_{4}+ 41.81107x_{1}x_{5}-0.774279x_{2}x_{3}+2374.4556x_{2}x_{4}-31.59058x_{2}x_{5}+13730.5476x_{3}x_{4}-213.701x_{3}x_{5}-4.81E^{05}x_{4}x_{5}+0.283245x_{1}^{2}+0.003566x_{2}^{2}+29.09881x_{3}^{2}+ 2.35E^{07}x_{4}^{2}+3112.13232x_{5}^{2}$ | (41) |
| --- | --- |

The accuracy and validation of the models are done based on the data in Table 5.

S1 Table 5. Fit statistics of the four models of the designed two-stage VCRS.

| **Index** | **EE** | **CAPEX** | **OPEX** | **GWP** |
| --- | --- | --- | --- | --- |
| R² | 0.995 | 0.998 | 0.999 | 0.999 |
| Adjusted R² | 0.986 | 0.994 | 0.996 | 0.996 |
| Predicted R² | 0.944 | 0.966 | 0.979 | 0.979 |
| Adeq Precision | 42.376 | 67.659 | 78.754 | 78.754 |
| Std. Dev. | 0.006 | 11.140 | 1.980 | 21.280 |
| Mean | 0.310 | 1571.190 | 142.600 | 1533.760 |
| CV % | 1.780 | 0.709 | 1.390 | 1.390 |
