# Supplementary material for: 4E analysis of a two-stage refrigeration system through surrogate models based on response surface methods and hybrid grey wolf optimizer
Source: PLoS One. 2023 Feb 3;18(2):e0272160. doi: 10.1371/journal.pone.0272160 (PMC9897521; doi:10.1371/journal.pone.0272160)
Supplement: S3 File — (DOCX) [file pone.0272160.s003.docx]

| 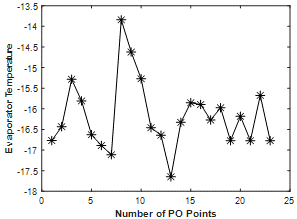 | 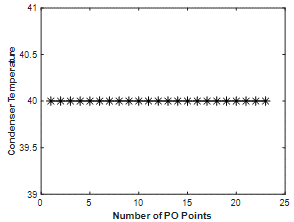 | 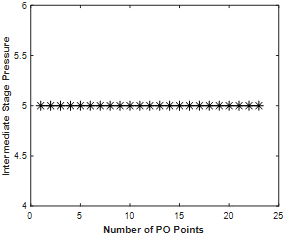 |
| --- | --- | --- |
| (a) | (b) | (c) |

## S3 File: Pareto characterization for the decision variables.

| 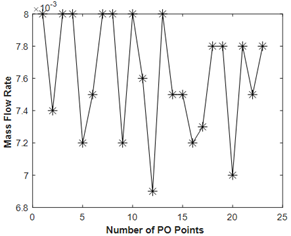 | 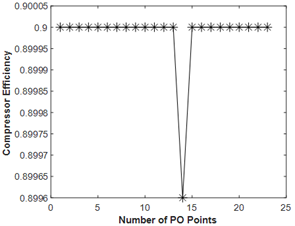 |
| --- | --- |
| (d) | (e) |

S3 Fig 1 (a-e): Cluster one of the pareto optimal solution sets of EE vs CAPEX.

| 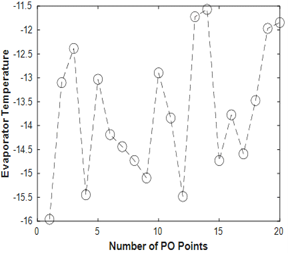 | 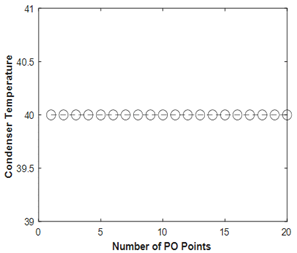 | 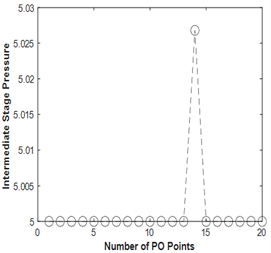 |
| --- | --- | --- |
| (a) | (b) | (c) |

| 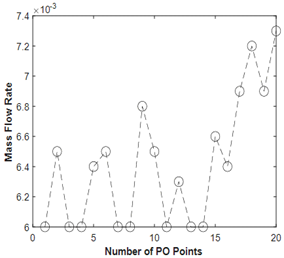 | 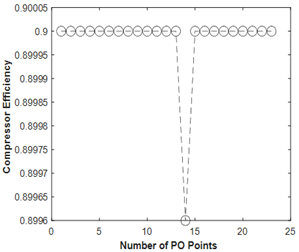 |
| --- | --- |
| (d) | (e) |

S3 Fig 2 (a-e): Cluster two of the pareto optimal solution sets of EE vs CAPEX.

| 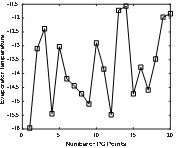 | 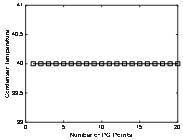 | 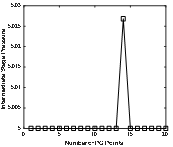 |
| --- | --- | --- |
| (a) | (b) | (c) |

| 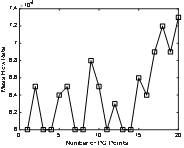 | 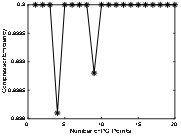 |
| --- | --- |
| (d) | (e) |

S3 Fig 3 (a-e): Cluster three of the pareto optimal solution sets of EE vs CAPEX.

| 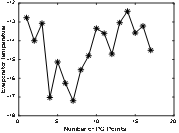 | 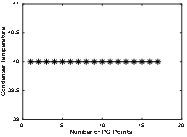 | 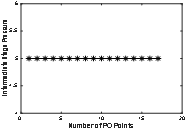 |
| --- | --- | --- |
| (a) | (b) | (c) |

| 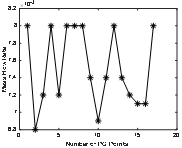 | 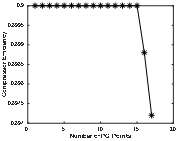 |
| --- | --- |
| (d) | (e) |

S3 Fig 4 (a-e): Cluster one of the pareto optimal solution sets of EE vs OPEX.

| 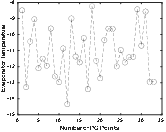 | 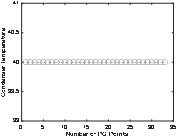 | 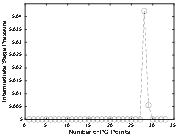 |
| --- | --- | --- |
| (a) | (b) | (c) |

| 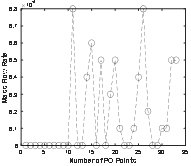 | 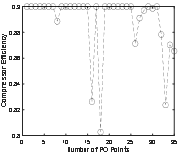 |
| --- | --- |
| (d) | (e) |

S3 Fig 5 (a-e): Cluster two of the pareto optimal solution sets of EE vs OPEX.

| 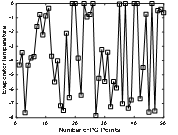 | 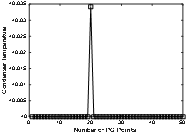 | 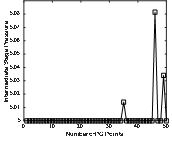 |
| --- | --- | --- |
| (a) | (b) | (c) |

| 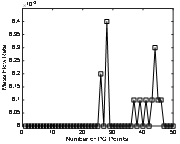 | 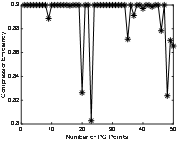 |
| --- | --- |
| (d) | (e) |

S3 Fig 6 (a-e): Cluster three of the pareto optimal solution sets of EE vs OPEX.

| 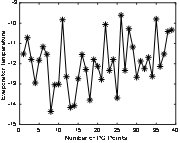 | 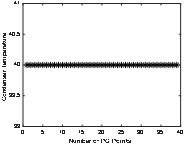 | 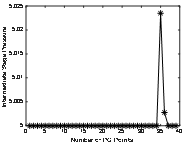 |
| --- | --- | --- |
| (a) | (b) | (c) |

| 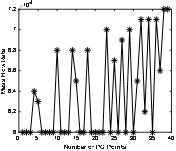 | 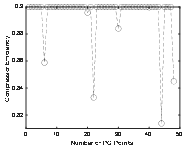 |
| --- | --- |
| (d) | (e) |

S3 Fig 7 (a-e): Cluster one of the pareto optimal solution sets of EE vs GWP.

| 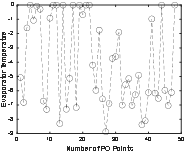 | 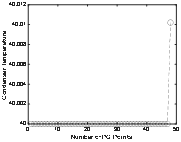 | 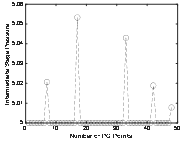 |
| --- | --- | --- |
| (a) | (b) | (c) |

| 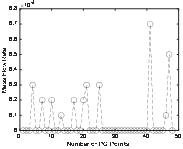 | 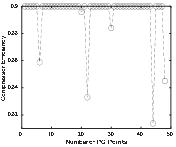 |
| --- | --- |
| (d) | (e) |

S3 Fig 8 (a-e): Cluster two of the pareto optimal solution sets of EE vs GWP.

| 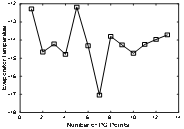 | 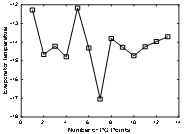 | 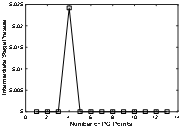 |
| --- | --- | --- |
| (a) | (b) | (c) |

| 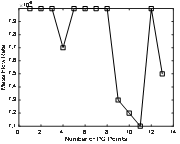 | 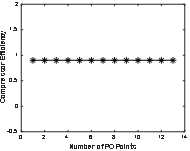 |
| --- | --- |
| (d) | (e) |

S3 Fig 9 (a-e): Cluster three of the pareto optimal solution sets of EE vs GWP.
